# Chemiluminescence Imaging of Superoxide Anion Detects Beta-Cell Function and Mass

**DOI:** 10.1371/journal.pone.0146601

**Published:** 2016-01-11

**Authors:** Laura L. Bronsart, Christian Stokes, Christopher H. Contag

**Affiliations:** 1 Department of Biology, Stanford University, 318 Campus Drive, Stanford, California 94305, United States of America; 2 Department of Pediatrics, Stanford University, 318 Campus Drive, Stanford, California 94305, United States of America; 3 Departments of Radiology, Microbiology & Immunology, Stanford University, 318 Campus Drive, Stanford, California 94305, United States of America; Albany Medical College, UNITED STATES

## Abstract

Superoxide anion is produced during normal cellular respiration and plays key roles in cellular physiology with its dysregulation being associated with a variety of diseases. Superoxide anion is a short-lived molecule and, therefore, its homeostatic regulation and role in biology and disease requires dynamic quantification with fine temporal resolution. Here we validated coelenterazine as a reporter of intracellular superoxide anion concentration and used it as a dynamic measure both *in vitro* and *in vivo*. Chemiluminescence was dependent upon superoxide anion levels, including those produced during cellular respiration, and concentrations varied both kinetically and temporally in response to physiologically relevant fluctuations in glucose levels. *In vivo* imaging with coelenterazine revealed that beta cells of the pancreas have increased levels of superoxide anion, which acted as a measure of beta-cell function and mass and could predict the susceptibility of mice to diabetes mellitus. Glucose response and regulation are key elements of cellular physiology and organismal biology, and superoxide anion appears to play a fundamental and dynamic role in both of these processes.

## Introduction

Reactive oxygen species, including superoxide anion, are highly reactive intermediates best known for their ability to induce cellular damage and are thought to play key roles in the pathogenesis of various chronic diseases including cancer, inflammation and aging [1–6]. They are primarily produced during normal cellular respiration and their quantity is tightly regulated by antioxidant scavenger enzymes. Although best known for their deleterious affects, reactive oxygen species are now being recognized as important intra- and intercellular signaling molecules with diverse functions ranging from cell-cell communication to cell division [7–15]. As such, reactive oxygen species play a fundamental role in cellular physiology and, therefore, biology.

Despite the significance of reactive oxygen species, such as superoxide anion, in both health and disease, there are currently few tools available for their study. Reporters of superoxide concentrations have largely been based on fluorescence, particularly hydrocyanine dyes, which have been optimized for microscopy and are, thus, limited to low-throughput *in vitro* or *ex vivo* studies. Additionally, these reporters fail to adequately provide a dynamic readout, particularly of reductions in superoxide concentrations over time [16–18]. Luminescent reporters circumvent some of the limitations of fluorescent reporters. One such chemiluminescent reporter, lucigenin, has been used to detect superoxide anion *in vitro* and *in vivo* [19]. However, its usefulness is limited due to its propensity to enhance superoxide anion formation [20–22]. The chemiluminescent superoxide anion probe L-012, a luminol analog, does not have this limitation, but, like lucigenin, it is restricted to the extracellular space [23,24]. As the majority of reactive oxygen species are produced within the cell and have very short-half lives, the ability to detect intracellular reactive oxygen species is necessary for their study in a biological context.

To begin to understand the role of superoxide anion in biology an imaging tool that can detect intra- and extracellular superoxide anion dynamically is required. Such an agent would enable the study of how superoxide anion concentrations change in response to various physiological and pathological states. We tested the luciferase substrate coelenterazine as such a tool. Coelenterazine is the small molecule substrate for Gaussia and Renilla luciferase enzymes. Because of its use in culture, it is known to be cell membrane permeable, chemiluminescent upon reaction with the reactive oxygen species superoxide anion and peroxynitrite and not to redox cycle like lucigenin [22,25–27]. As such, we hypothesized that coelenterazine could be applied to reveal novel insights into superoxide anion biology *in vitro* and *in vivo* in response to various physiological and pathological conditions.

## Results

Although coelenterazine is known to react with superoxide anion in chemical systems, its utility in quantifying biological superoxide anion was relatively unknown [25]. Previously, coelenterazine had been used to detect superoxide anion produced during the neutrophilic oxidative burst, demonstrating that it could be used to detect high concentrations released into the extracellular space [26]. Additionally, it was used to quantify the superoxide anion produced by the electron transport chain from purified mitochondria [20]. Although insightful, these studies were limited by highly artificial experimental designs. We aimed to quantify the dynamic variation in superoxide anion concentrations in a biological context, both *in vitro* and *in vivo*. To do this, we first began by determining if cells produce coelenterazine-mediated chemiluminescence under static conditions in a detectable and reproducible manner.

### Coelenterazine-mediated cellular chemiluminescence is detectable and can be quantified *in vitro*

**Fig 1:**
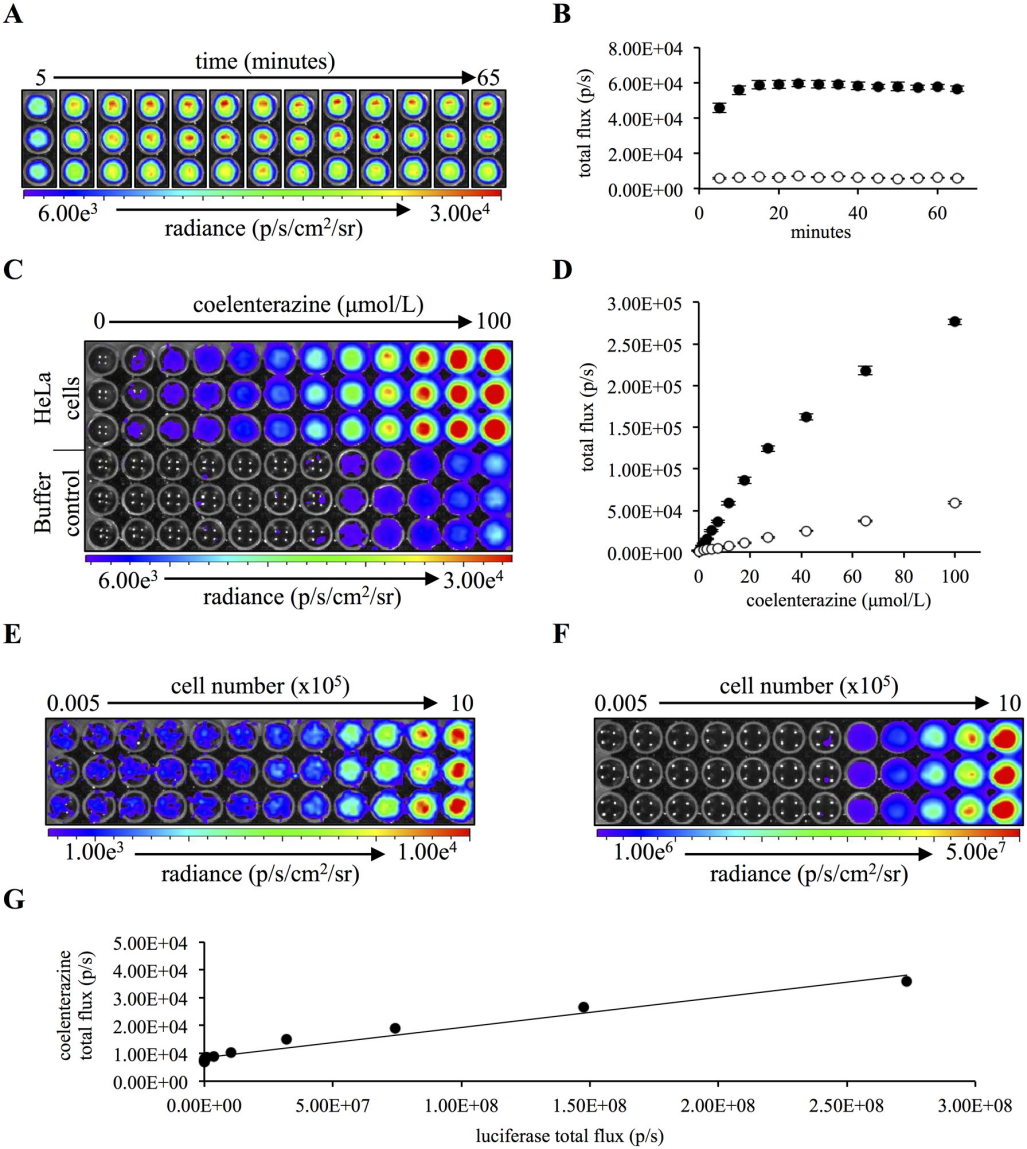
HeLa cells produce coelenterazine chemiluminescence *in vitro*. (A) 5x10^5^ HeLa cells were imaged sequentially, at 5 min intervals for 1 hour. (B) Quantification of HeLa (black) or buffer (white) coelenterazine chemiluminescence at each time point (n = 3). (C) 5x10^5^ HeLa cells were imaged at 15 minutes following the addition of native coelenterazine ranging in concentration from 0 μmol/L to 100 μmol/L. (D) Quantification of HeLa (black) or buffer (white) coelenterazine chemiluminescence (n = 3). (E) A range of 500 to 1x10^6^ HeLa cells were imaged 15 minutes following the addition of 10 μmol/L of native coelenterazine. (F) A range of 500 to 1x10^6^ HeLa cells were imaged 1 minute following the addition of 1.07 mmol/L luciferin. (G) Correlation graph of coelenterazine chemiluminescent signal intensity (y-axis) versus the corresponding luciferase signal intensity (x-axis) for a given cell concentration. (Error bars represent the mean ± SEM.)

We used the HeLa cervical-cancer cell line to characterize cellular coelenterazine-mediated chemiluminescence *in vitro*. To test if non-immunological cells would produce coelenterazine-mediated chemiluminescence under basal conditions, 5x10^5^ HeLa cells or a buffer control were plated in triplicate, and 11.6 μmol/L of native coelenterazine was added to each well. Chemiluminescence was detected using a cooled CCD camera (IVIS). Significant cellular chemiluminescence was detected within 5 minutes post-coelenterazine addition (P<0.0001), reached a maximum intensity, 8.6 ± 0.4 (mean ± SEM) fold above background, within 15-minutes post-addition and maintained that signal intensity for the remaining 45 minutes of imaging (Fig 1A and 1B). These results indicated that non-immunological cells in static conditions do stimulate coelenterazine chemiluminescence.

We next assessed if the cellular chemiluminescence was coelenterazine-concentration dependent. HeLa cells were treated with a range of coelenterazine concentrations from 0 μmol/L to 100 μmol/L. There was a significant, positive correlation (R^2^ = 0.9617; P < 0.0001) between the coelenterazine concentration and signal intensity (Fig 1C and 1D). The cellular chemiluminescence was significantly greater than the buffer control at a concentration of 1.3 μmol/L (P = 0.0079) and at each higher concentration thereafter. No plateau in signal intensity was observed, even at the highest coelenterazine concentrations. The dose sensitivity and the absence of a plateau in signal intensity emphasized the necessity for stringent intra-experimental controls and the limitation of inter-experimental interpretation.

Having determined that HeLa cells do produce coelenterazine chemiluminescence in a concentration-dependent manner, we next investigated if the chemiluminescent signal intensity correlated with cell number. A range of 500 to 1x10^6^ luciferase-transfected HeLa cells was imaged for chemiluminescence in the presence of coelenterazine, and bioluminescence in the presence of the luciferase substrate, luciferin. It had been previously demonstrated that luciferase-transfected cell lines produce a bioluminescent signal whose intensity correlates with cell number [28]. Both the coelenterazine and luciferase signals demonstrated a positive correlation with cell number (Fig 1E and 1F) with a significant correlation (P < 0.0001) between the two imaging agents, R^2^ = 0.9634 (Fig 1G). As expected, the luciferase signal was much greater and detected fewer than 500 HeLa cells; the coelenterazine signal was less intense and required greater than 15,000 cells to achieve significant signal over the buffer control. The lower signal from coelenterazine is due to both a lower light output and a higher background signal. The lower signal intensity is likely a consequence of fewer reactants of lesser efficiency being available in a given cell when compared to a cell that has been stably transfected with a luciferase gene and expressing high levels of the enzyme [29]. The high background signal is a consequence of the high auto-oxidation rate of coelenterazine in solution [30]. Despite these limitations, collectively, these data indicate that HeLa cells stimulate the oxidation of coelenterazine to coelenteramide with the resultant photon emission being detectable in low-light imaging systems.

### Coelenterazine chemiluminescence is mediated by reaction with superoxide anion produced during cellular respiration

**Fig 2:**
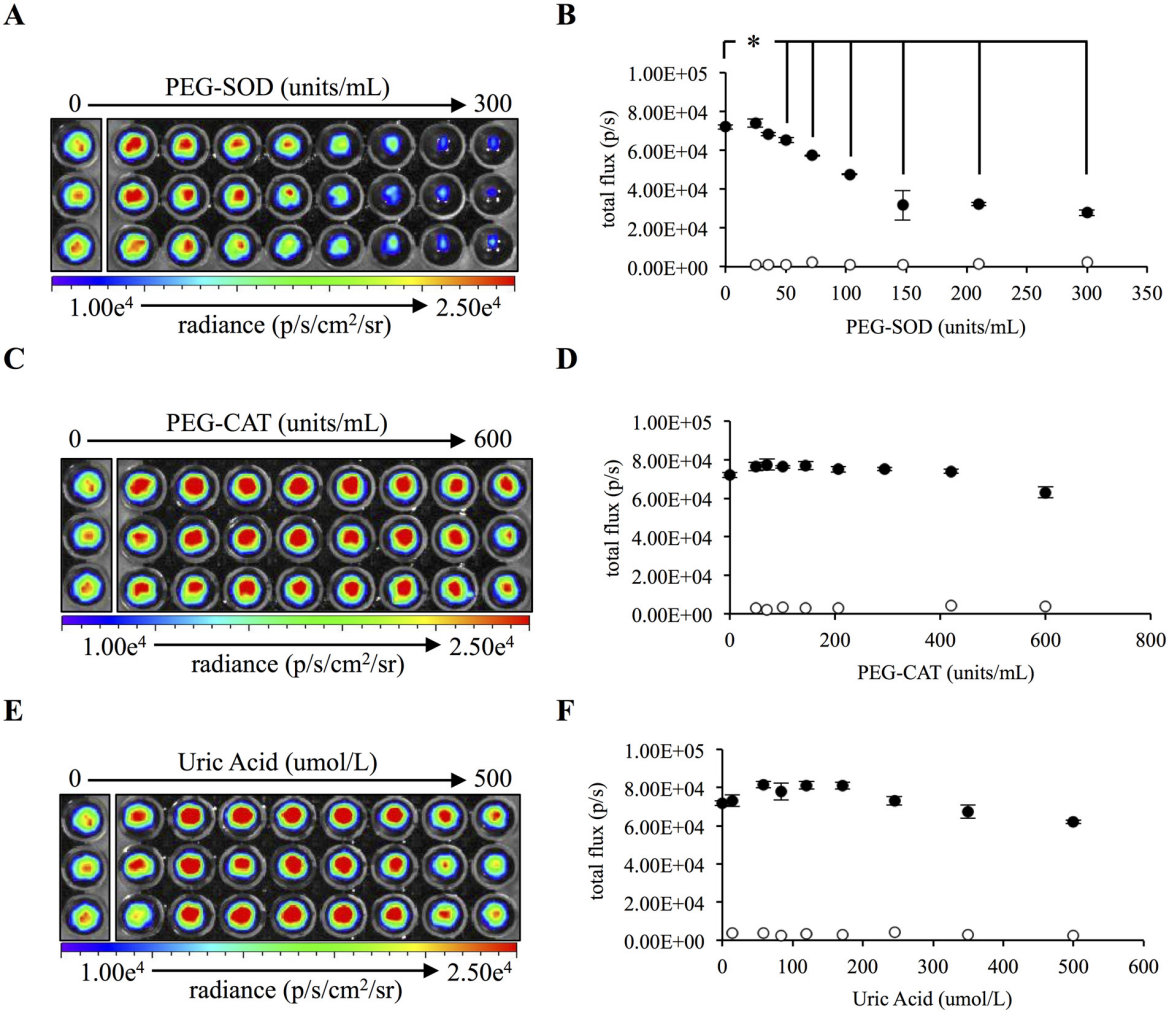
Coelenterazine chemiluminescence is dependent upon superoxide anion concentrations *in vitro*. (A) HeLa cells were treated with range of 0 units/mL to 300 units/mL of PEG-SOD and imaged sequentially. The image was taken at 95 minutes post PEG-SOD addition. (B) Quantification of HeLa (black) or buffer (white) coelenterazine chemiluminescence for each concentration 95 minutes post PEG-SOD addition (n = 3). (C) HeLa cells were treated with range of 0 units/mL to 600 units/mL of PEG-CAT and imaged sequentially. Image is 95 minutes post PEG-CAT addition. (D) Quantification of HeLa (black) or buffer (white) coelenterazine chemiluminescence for each concentration 95 minutes post PEG-CAT addition (n = 3). (E) HeLa cells were treated with range of 0 μmol/L to 500 μmol/L of uric acid and imaged sequentially. Image is 95 minutes post uric acid addition. (F) Quantification of HeLa (black) or buffer (white) coelenterazine chemiluminescence for each concentration 95 minutes post uric acid addition (n = 3). (Error bars represent the mean ± SEM, and * denotes significant differences of P < 0.02.)

Coelenterazine is known to react with the reactive oxygen species superoxide anion and peroxynitrite with resultant photon emission but does not react with hydrogen peroxide [22,26]. To determine if coelenterazine was reacting with cellular reactive oxygen species and to verify these earlier studies, HeLa cells were treated with reactive oxygen species scavengers and imaged for coelenterazine-mediated chemiluminescence. Incubation with pegylated superoxide dismutase (PEG-SOD), a superoxide anion scavenger, resulted in a significant 55.0 ± 2.0% (mean ± SEM) decrease in signal intensity (Fig 2A and 2B). In contrast, pegylated catalase (PEG-CAT), a hydrogen peroxide scavenger, had no significant affect (Fig 2C and 2D). Similarly, uric acid, a cell-permeable scavenger for peroxynitrite, had little to no affect (Fig 2E and 2F). These findings suggested that the reaction of coelenterazine with superoxide anion was the major contributor to the detected chemiluminescent signal.

**Fig 3:**
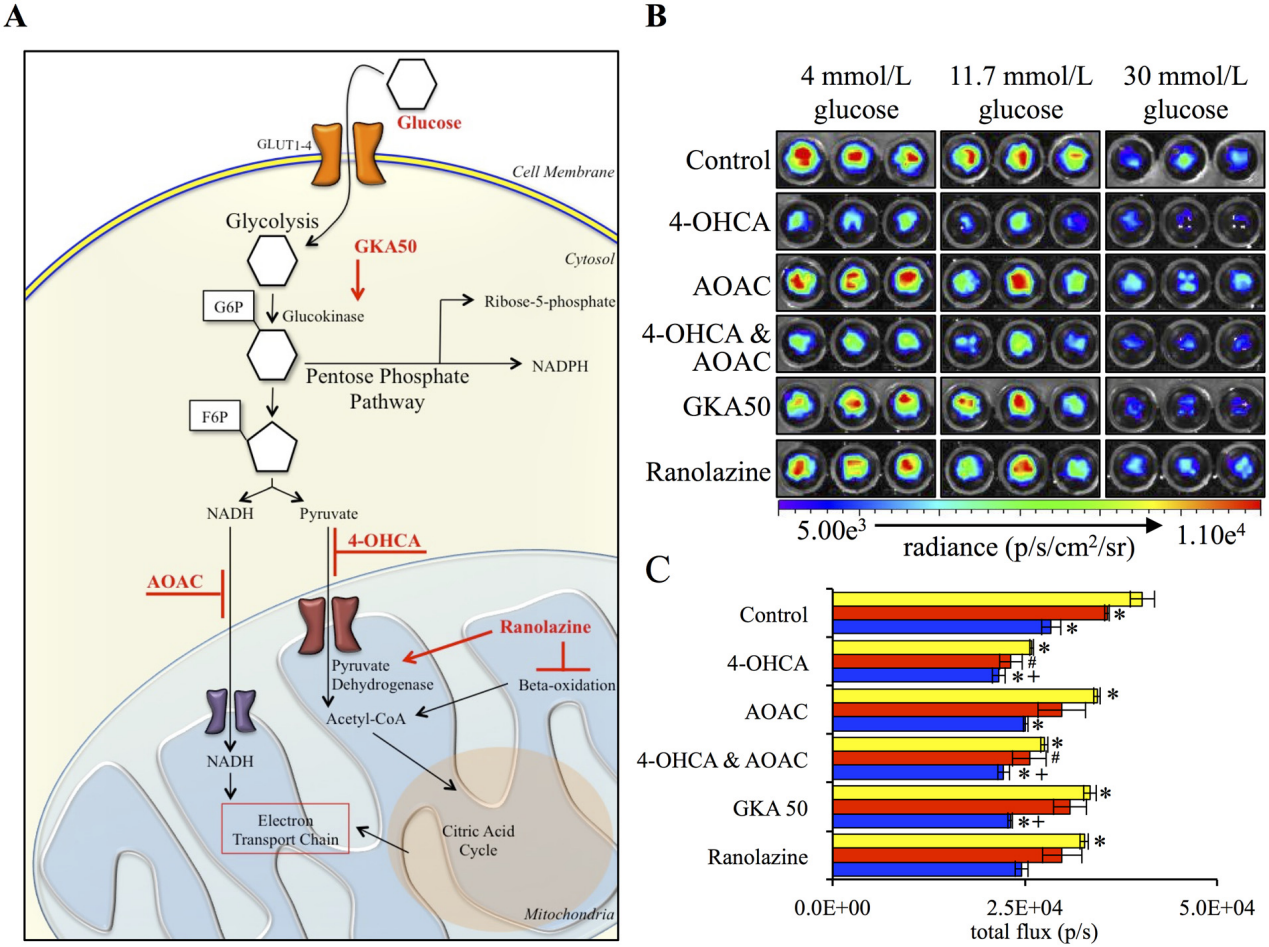
Treatment with mitochondrial shuttle inhibitors, GKA50, Ranolazine and glucose results in significant decreases in superoxide concentration as detected by coelenterazine chemiluminescence. (A) Bold, red font indicates methods of perturbation. The physiological response of cells was assessed by addition of glucose, GKA50, an activator of glycolysis, AOAC, an inhibitor of the malate-aspartate shuttle, 4-OHCA, an inhibitor of the pyruvate and lactate shuttle, ranolazine, an inhibitor of beta-oxidation and an activator of pyruvate dehydrogenase. (B) HeLa cells were treated with vehicle control, 250 μmol/L of 4-OHCA, 100 μmol/L AOAC, a combination of 4-OHCA and AOAC, 1 μmol/L GKA50 or 1 μmol/L Ranolazine and the final glucose concentrations adjusted from 4 mmol/L to 4 mmol/L, 11.7 mmol/L or 30 mmol/L and sequentially imaged. Representative image is 95 minutes post treatment. (C) Quantification of coelenterazine chemiluminescence 95 minutes post-treatment (n = 3). (Error bars represent the mean ± SEM, * denotes a significant difference from the 4 mmol/L control of P < 0.05, # denotes a significant difference from the 11.7 mmol/L control of P < 0.015, and + denotes a significant difference from the 30 mmol/L control of P < 0.015.)

It has been previously demonstrated that the majority of cellular superoxide anion is produced by the mitochondria during oxidative phosphorylation [31–33]. To further support the detection of superoxide anion and to verify its origins, HeLa cells were treated with activators or inhibitors of cell metabolic pathways (Fig 3A). Treatment with the pyruvate and lactate shuttle inhibitor 4-OHCA resulted in 35.6 ± 2.3% (mean ± SEM) decrease in signal intensity. The malate-aspartate shuttle inhibitor AOAC resulted in a decrease of 14.3 ± 3.6% (mean ± SEM). These findings are consistent with previous work that demonstrated that inhibition of the pyruvate shuttle resulted in a greater reduction in mitochondrial superoxide anion production than did inhibition of the malate-aspartate shuttle [34]. Addition of both shuttle inhibitors had a similar affect on signal intensity as treatment with 4-OHCA alone. Treatment with GKA50, an activator of glycolysis, resulted in a modest, but significant, reduction in signal intensity. Additionally, HeLa cells were treated with Ranolazine, an activator of pyruvate dehydrogenase and inhibitor of fatty acid beta-oxidation [35]. Similar to GKA50 treatment, Ranolazine caused a modest, but significant drop in the signal intensity of 18.4 ± 3.3% (mean ± SEM). The addition of glucose at concentrations of 11.7 mM and 30 mM also resulted in a significant decrease in superoxide anion concentrations as quantified by coelenterazine chemiluminescence. The glucose-mediated decrease was not inhibited by the presence of mitochondrial shuttle inhibitors and, in fact, there was an additive effect when combined with these reagents (Fig 3B and 3C). These results may indicate that the mechanism for the glucose-mediated reduction in superoxide anion concentrations occurs independent of changes in mitochondrial activity; however, further experimentation using additional measures of mitochondrial activity are necessary to verify these findings.

### Acute exposure to physiological glucose concentrations results in dynamic changes in cellular superoxide anion concentration

**Fig 4:**
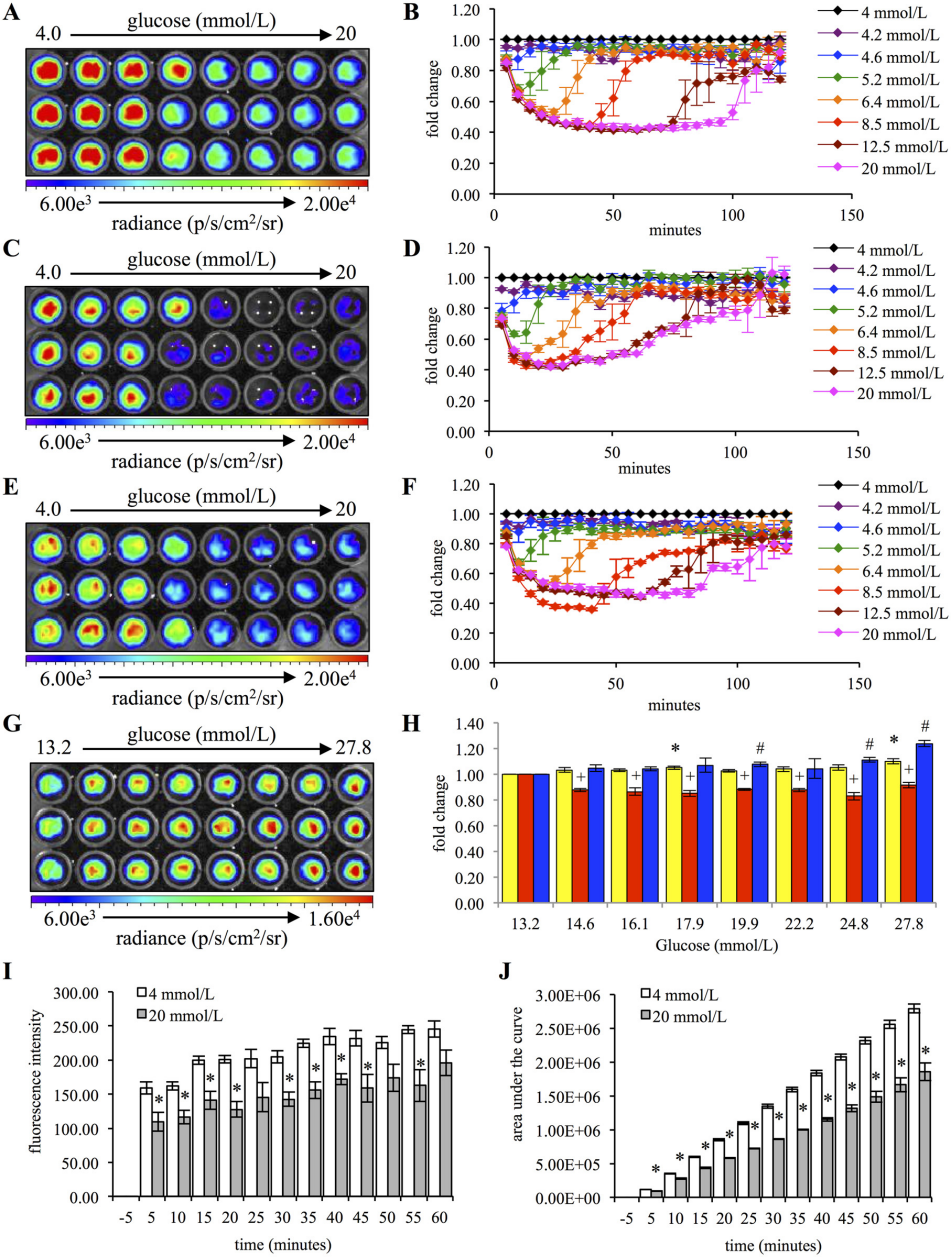
Acute hyperglycemia results in dynamic changes in the superoxide anion concentration of HeLa cells. (A) 5x10^5^ HeLa cells were exposed to glucose concentrations ranging from 4 mmol/L (control) to 20 mmol/L and sequentially imaged. Representative image is 15 minutes post treatment. (B) Quantification of coelenterazine chemiluminescence at each time point for each sample (n = 3). (C) 5x10^5^ RIN-5F cells were exposed to glucose concentrations ranging from 4 mmol/L (control) to 20 mmol/L and sequentially imaged. Representative image is 15 minutes post treatment. (D) Quantification of coelenterazine chemiluminescence at each time point for each sample (n = 3). (E) 5x10^5^ INS-1 cells were exposed to glucose concentrations ranging from 4 mmol/L (control) to 20 mmol/L and sequentially imaged. Representative image is 15 minutes post treatment. (F) Quantification of coelenterazine chemiluminescence at each time point for each sample (n = 3). (G) 5x10^5^ HeLa cells were exposed to glucose concentrations ranging from 13.2 mmol/L (control) to 27.8 mmol/L and sequentially imaged. Representative image is 120 minutes post treatment. (H) Quantification of coelenterazine chemiluminescence for HeLa, RIN-5F and INS-1 cells treated as described in G at 120 minutes post-treatment (n = 3). (Error bars represent the mean ± SEM, * denotes a significant difference from the 13.2 mmol/L HeLa cells of P < 0.015, + denotes a significant difference from the 13.2 mmol/L RIN-5F cells of P < 0.015, and # denotes a significant difference from the 13.2 mmol/L INS-1 cells of P < 0.015.) (I) Dihydroethidium assay of 5x10^5^ HeLa cells exposed to glucose concentrations of 4 mmol/L (control) or 20 mmol/L with the quantification of 2-hydroxyethidium fluorescence as each time point minus the pre-treatment (-5 minutes) fluorescence intensity for each sample (n = 3). (Error bars represent the mean ± SEM, * denotes a significant difference from the 4 mmol/L control of P < 0.04.) (J) Coelenterazine assay of 5x10^5^ HeLa cells exposed to glucose concentrations of 4 mmol/L (control) or 20 mmol/L with the quantification as the cumulative area under the curve at each time point minus the pre-treatment (-5 minutes) chemiluminescence for each sample (n = 3). (Error bars represent the mean ± SEM, * denotes a significant difference from the 4 mmol/L control of P < 0.004.)

The previous studies determined that coelenterazine could be used to detect mitochondrial superoxide anion production in non-immunological cells, and that it could be used to monitor changes in superoxide anion concentrations in response to acute stimuli. The result that hyperglycemia caused a reduction in superoxide anion concentrations is contrary to many reports in which hyperglycemia results in an increase in reactive oxygen species including superoxide anion [34,36–39]. As such, we aimed to further characterize the acute response of superoxide anion concentrations to physiologically relevant normoglycemic and hyperglycemic conditions. To this end, HeLa cells were sequentially imaged with coelenterazine following adjustment of the glucose concentration in the imaging buffer from 4mmol/L up to 20 mmol/L. Within 15 minutes following the addition of glucose, a significant decrease was observed for glucose concentrations equal to, or greater than, 5.2 mmol/L (Fig 4A). The higher the concentration of glucose, the greater the decrease and the longer that decrease was maintained. There was a maximal signal decrease of 58.6 ± 1.0% (mean ± SEM). For cells exposed to 12.5 mmol/L and 20 mmol/L glucose, this signal intensity was maintained as a plateau for approximately 40 to 60 minutes respectively. Within 2 hours following glucose addition, the signal intensities for all samples had returned to or were near the 4 mmol/L baseline (Fig 4B).

To determine if this phenomenon was unique to the human HeLa cell line, we tested two additional pancreatic beta-cell lines, RIN-5F and INS-1. The RIN-5F cell line was more sensitive to glucose, demonstrating a significant decrease at a glucose concentration of 4.6 mM (Fig 4C). Despite this increased sensitivity, the RIN-5F cell line signal intensity returned to the 4 mmol/L baseline more rapidly than either the HeLa or INS-1 cell lines (Fig 4D). The INS-1 cell line was less sensitive, decreasing significantly at concentrations of 6.4 mmol/L or greater (Fig 4E*)*. Additionally, it demonstrated similar imaging kinetics as the HeLa cell line (Fig 4F).

Based on our findings that the cellular response to glucose is a dynamic process, we hypothesized that exposure to higher glucose concentrations that were more consistent with prior literature may result in an increase in superoxide anion concentrations and, therefore, an increase in chemiluminescence. To test this, HeLa, RIN-5F and INS-1 cells were exposed to glucose concentrations ranging from 13.2 mmol/L to 28.7 mmol/L and imaged. At 120 minutes following glucose addition, the HeLa cell line exposed to 27.8 mmol/L glucose concentration had a significantly higher chemiluminescent signal than HeLa cells exposed to 13.2 mmol/L (Fig 4G). Additionally, INS-1 cells exposed to 24.8 mmol/L or greater glucose concentrations had a significantly higher signal than cells maintained at 13.2 mmol/L glucose. The RIN-5F cell line did not have a similar increase in signal (Fig 4H).

To validate our findings, we completed the same glucose experiment using both coelenterazine and a common superoxide anion reporter, dihydroethidium, in parallel. To enable comparison between the two reporters, the coelenterazine data was computed as the cumulative area under the curve for each time point. When viewed in this manner, both reporters demonstrated significantly less signal intensity from cells treated with 20 mmol/L glucose versus those maintained at 4 mmol/L glucose (Fig 4I and 4J). Additionally, it was evident that dihydroethidium, a fluorescent probe that accumulates signal over time, had a limited dynamic range. Despite the reduced dynamic range of dihydroethidium, the two reporters had a significant (P < 0.0001) positive correlation (R^2^ = 06753) (S1 Fig).

### *In vivo* imaging with coelenterazine detects superoxide anion of pancreatic origin in healthy mice

**Fig 5:**
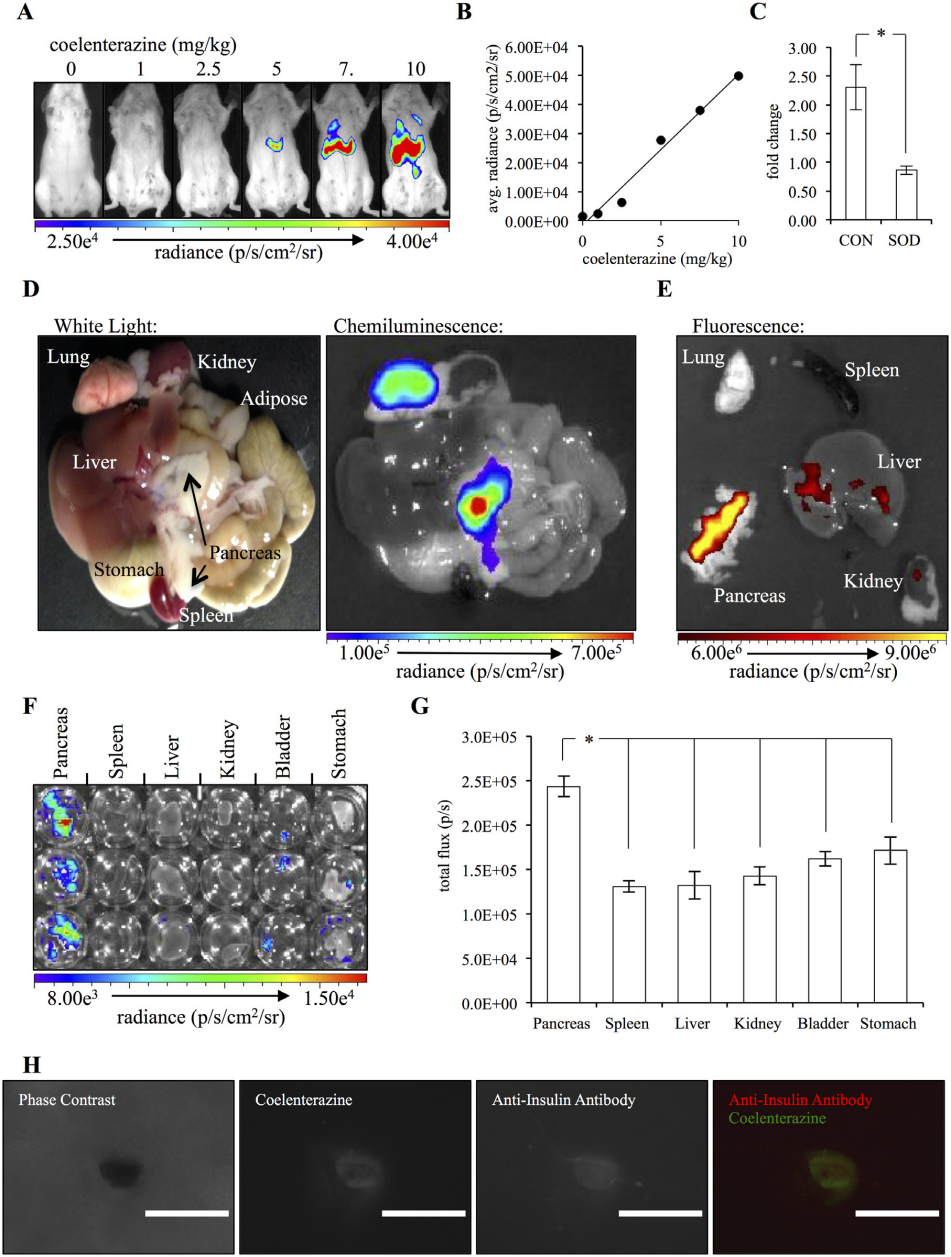
Coelenterazine administration *in vivo* results in chemiluminescent detection of superoxide anion in a dose-dependent manner that is of pancreatic origin. (A) Healthy mice were administered 0, 1, 2.5, 5, 7.5 and 10 mg/kg coelenterazine and imaged for chemiluminescence. (B) Quantification of the chemiluminescent signal for each coelenterazine dose (n = 1). (C) The fold change in the abdominal chemiluminescent signal from before to 20 hours following treatment with 600 units of PEG-SOD (SOD) or vehicle control (CON) (n = 3). (D) Tissues collected from a mouse euthanized 3 minutes post-administration of 5 mg/kg native coelenterazine. (E) Tissues from the mouse in D fluorescently imaged with an excitation of 430 nm and emission of 520 nm. (F) Isolated tissues treated with 20 μg of native coelenteraine *ex vivo*. (G) Quantification of the chemiluminescent signal from each well (n = 3). (Error bars represent the mean ± SEM, and * denotes a significant difference of P < 0.02.) (H) Whole mouse pancreas was excised and incubated in 20 μmol/L coelenterazine, fixed and stained with anti-insulin antibody followed by an Alexa Fluor 750 secondary. (Scale bar represents 400 μm.)

Coelenterazine has been used *in vivo* for bioluminescence imaging as the substrate for several luciferase enzyme including *Gaussia* and *Renilla*; however, to our knowledge, it had not been evaluated, alone, as an indicator of superoxide anion concentrations *in vivo* [40–42]. As such, we first aimed to characterize the *in vivo* chemiluminescence produced following coelenterazine injection in the absence of the catalyzing enzymes *Gaussia* or *Renilla*. Coelenterazine was administered to healthy mice at varying doses and the resulting photon emission detected using an IVIS Spectrum. Injection of coelenterazine resulted in an upper-abdominal signal and the intensity increased with increases in the dose of coelenterazine (Fig 5A and 5B). The abdominal signal was greatest at 1 to 5 minutes post-injection and then declined. Approximately 5 minutes post-injection, a thoracic signal from the lung became the dominant region of chemuliminescence (S2A and S2B Fig). We focused on the abdominal region for all future studies for two reasons: first, its decrease prior to depletion of circulating coelenterazine, as indicated by the prolonged lung signal, suggested a tissue-specific mechanism of chemiluminescence and, secondly, both isoflurane and 100% oxygen are known to induce oxidative stress and this was our method of anesthesia [43,44]. Multiple coelenterazine analogs resulted in the upper-abdominal chemiluminescent signal (S3A and S3B Fig). Additionally, the *in vivo* delivery of PEG-SOD resulted in a significant decrease in the signal 20 hours following treatment relative to vehicle control-treated animals (P = 0.02251), suggesting that the *in vivo* signal was, at least partly, the result of superoxide anion (Fig 5C). Imaging mice periodically over the course of 24 hours revealed, although not statistically significant, a circadian signal pattern with a peak in signal intensity occurring midway through the mouse sleep phase (S4A and S4B Fig). Additionally, fasted mice showed reduced signal intensity (S5A and S5B Fig). Collectively these findings suggest that the abdominal chemiluminescent signal is partially mediated by superoxide anion production and that its concentration is dependent upon environmental cues.

Having established that coelenterazine administration in healthy mice can be used to detect superoxide anion production, we next aimed to determine the tissue of origin. Following intravenous administration of coelenterazine, a mouse was euthanized and its tissues immediately imaged. *Ex vivo* imaging revealed prominent chemiluminescence coming from both the pancreas and lung (Fig 5D). This was consistent with our *in vivo* imaging that showed an abdominal and thoracic signal. Additionally, having established the absorption and emission sepctra of the coelenterazine-reaction by-product coelenteramide (S6A and S6B Fig), we fluorescently imaged the tissues. Coelenteramide presence was confirmed in the pancreas but not the lung (Fig 5E). The fluorescent signal was not detection of coelenterazine chemiluminescence emission through the 520/10 nm emission filter or a consequence of pancreatic autofluorescence (S7A–S7C Fig). This result supported that the pancreatic-coelenterazine reaction was likely intracellular versus occurring within the circulation associated with the tissue. To further confirm the specificity of the pancreatic chemiluminescent signal, tissues were collected from three mice and 20 μg of coelenterazine added *ex vivo* (Fig 5F). Even in the absence of vascular delivery, the pancreas had significantly greater chemiluminescent signal than all other tissues evaluated (Fig 5G). Furthermore, microscopic evaluation of whole pancreas incubated with 20 μmol/L of coelenterazine, fixed, and then incubated with anti-insulin antibody showed colocalization of the coelenteramide fluorescence and anti-insulin antibody, suggesting the coelenterazine reaction occurs at a greater rate in the specialized endocrine portion of the pancreas, the islets of Langerhans (Fig 5H).

The dominant *in vivo* pancreatic signal and the *ex vivo* islet of langerghans fluorescence was interesting because the beta cells of the pancreas, the insulin-secreting cells, have an unusually low expression of some scavenger enzymes [45–47]. Additionally, beta cells utilize mitochondrial-derived reactive oxygen species as signaling molecules during the process of insulin secretion [48,49]. As the beta cells make up approximately 1.7% of the total pancreatic volume, it can be postulated that their concentration of superoxide anion would need to be significantly elevated relative to other tissues to achieve a detectable coelenterazine signal [50].

### Beta-cell superoxide anion concentrations are greater than other tissues and can be detected using coelenterazine

**Fig 6:**
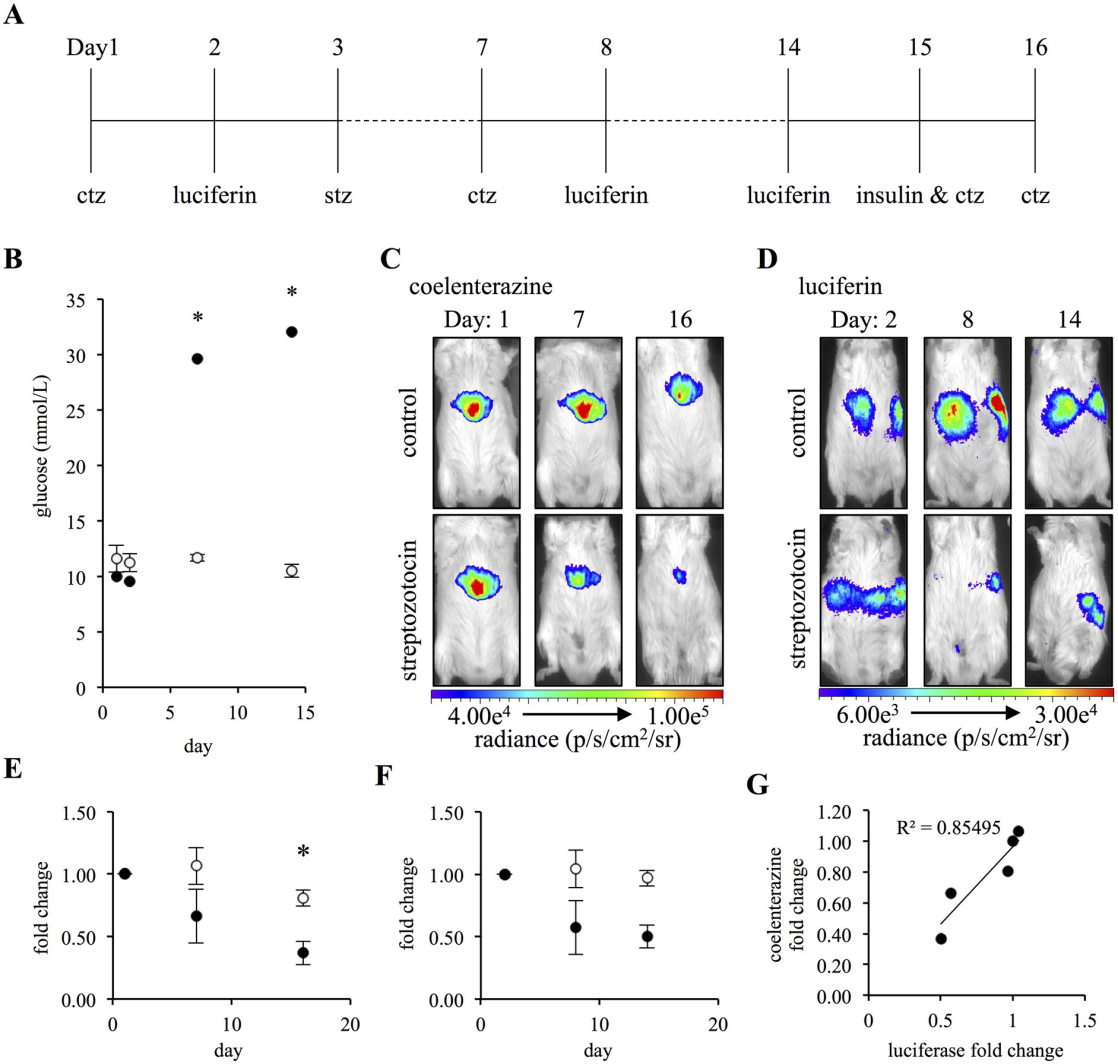
Coelenterazine chemiluminescence correlates with a loss of functional beta-cell mass in the MIP-Luc-VU. (A) Schematic representation of the study design. (B) Blood glucose concentrations of vehicle control (white) and streptozotocin (black) treated mice (n = 4). (C) Images of a representative control and streptozotocin mouse imaged with coelenterazine. (D) Images of the animals in C imaged with luciferin. (E) Fold change in coelenterazine signal of vehicle control (white) and streptozotocin (black) treated mice (n = 4). (F) Fold change in luciferase signal of vehicle control (white) and streptozotocin (black) treated mice (n = 4). (G) Fold change in coelenterazine signal graphed against the corresponding fold change in luciferase signal. (Error bars represent the mean ± SEM, and * denotes a significance difference between the group of P < 0.0013.)

We utilized the streptozotocin mouse model to determine if the high superoxide anion concentrations detected *in vivo* were originating from pancreatic beta-cells. Application of the transgenic MIP-Luc-VU mouse, which expresses the firefly luciferase bioluminescent enzyme driven by the mouse insulin I promoter, enabled *in vivo* dual bioluminescent and chemiluminescent imaging with the bioluminescent luciferase reporter serving as an internal control for beta-cell mass (Fig 6A) [51]. Glucose monitoring confirmed that streptozotocin-treated mice developed clinical type I diabetes, which occurs when there is an approximate 90% reduction in beta-cell mass (Fig 6B) [52]. There was a reduction in signal intensity over time in the streptozotocin-treated mice when imaged with either coelenterazine or luciferin (Fig 6C and 6D). The streptozotocin-treated mice showed a significant (P = 0.0202) decrease of 34 ± 11% (mean ± SEM) in coelenterazine signal intensity from day 1 to 7. By day 16, the coelenterazine signal had significantly decreased (P = 0.0327) by an additional 29% for a significant (P = 9.49x10^-6^) total reduction of 63 ± 5% (mean ± SEM). The control group had significantly greater coelenterazine signal intensity when compared to the streptozotocin-treated group on day 16 (P = 0.0013). The control group did not demonstrate a significant difference in coelenterazine signal intensity over time (Fig 6E). Similarly, the streptozotocin-treated mice demonstrated a significant (P = 0.0060) 43 ± 10% reduction in luciferase activity from day 2 to 8 of imaging. There was an additional 7% decrease between days 8 and 14, resulting in a significant (P = 0.0093) total decrease of 50 ± 13% over the study period. The difference in luciferase activity between the control and streptozotocin-treated mice on day 14 of imaging was nearly significant (P = 0.0637). The control group did not demonstrate a significant difference in luciferase signal intensity over time (Fig 6F). The fold changes in the coelenterazine and luciferase signal intensities significantly correlated (P = 0.024; R^2^ = 0.8546) (Fig 6G). To verify that the reduction in coelenterazine signal intensity observed in the streptozotocin-treated group was not a consequence of imaging during hyperglycemia, diabetic mice were administered insulin immediately prior to coelenterazine imaging on day 15. When compared to coelenterazine images taken during hyperglycemia on day 16, there was no significant difference in coelenterazine signal intensity despite insulin therapy resulting in a significant (P = 0.0002) decrease in blood glucose levels (S8A and S8B Fig).

Collectively these results support that *in vivo* delivery of coelenterazine detects physiological levels of superoxide anion, that levels of superoxide anion are greatest in beta cells and that this characteristic results in a preferentially greater chemiluminescent signal from the pancreas. The knowledge that coelenterazine detected a decrease in beta-cell mass as a result of acute beta-cell, streptozotocin toxicity, led us to question if superoxide anion concentrations may serve as a biomarker for autoimmune type I diabetes.

### *In vivo* superoxide anion imaging of beta-cell mass can predict diabetes mellitus in the NOD mouse model

Type I diabetes is an autoimmune disease which results the destruction of the insulin-secreting cells, beta cells. Type II diabetes is the result of beta-cell dysfunction and loss as a consequence of insulin resistance. Clinically, a non-invasive technique for quantifying beta-cell mass may provide an indicator for early interventional therapies. Currently, diagnosis is primarily based on the development of hyperglycemia-associated clinical signs which do not arise until approximately 90% of the beta-cell mass has been destroyed [52].

To test if coelenterazine imaging of superoxide anion could be used to predict susceptibility to diabetes mellitus we employed the non-obese diabetic (NOD) mouse model. The NOD mouse-model is the predominant mouse model for type I diabetes. It is characterized by spontaneous inflammation of the islets of langerhans, insulitis, destruction of the pancreatic beta-cells and development of hyperglycemia. Although all mice appear to develop some degree of insulitis, not all mice develop hyperglycemia [53]. In addition to the spontaneous development and incomplete penetrance, the age of disease onset is highly variable. These parameters are influenced by unknown environmental factors [54]. The heterogeneity of this model makes its study particularly challenging; however, it also makes it the most accurate model for type I diabetes, which is also highly heterogeneous in humans [55]. For our purposes, it is this heterogeneity that made it the most suitable model to test if superoxide anion concentrations, as detected by coelenterazine *in vivo*, could predict clinical type I diabetes. To apply this model, NOD mice were obtained at 5 weeks of age, an age prior to the reduction of beta-cell mass [56]. The mice were imaged weekly with coelenterazine and blood glucose levels monitored.

**Fig 7:**
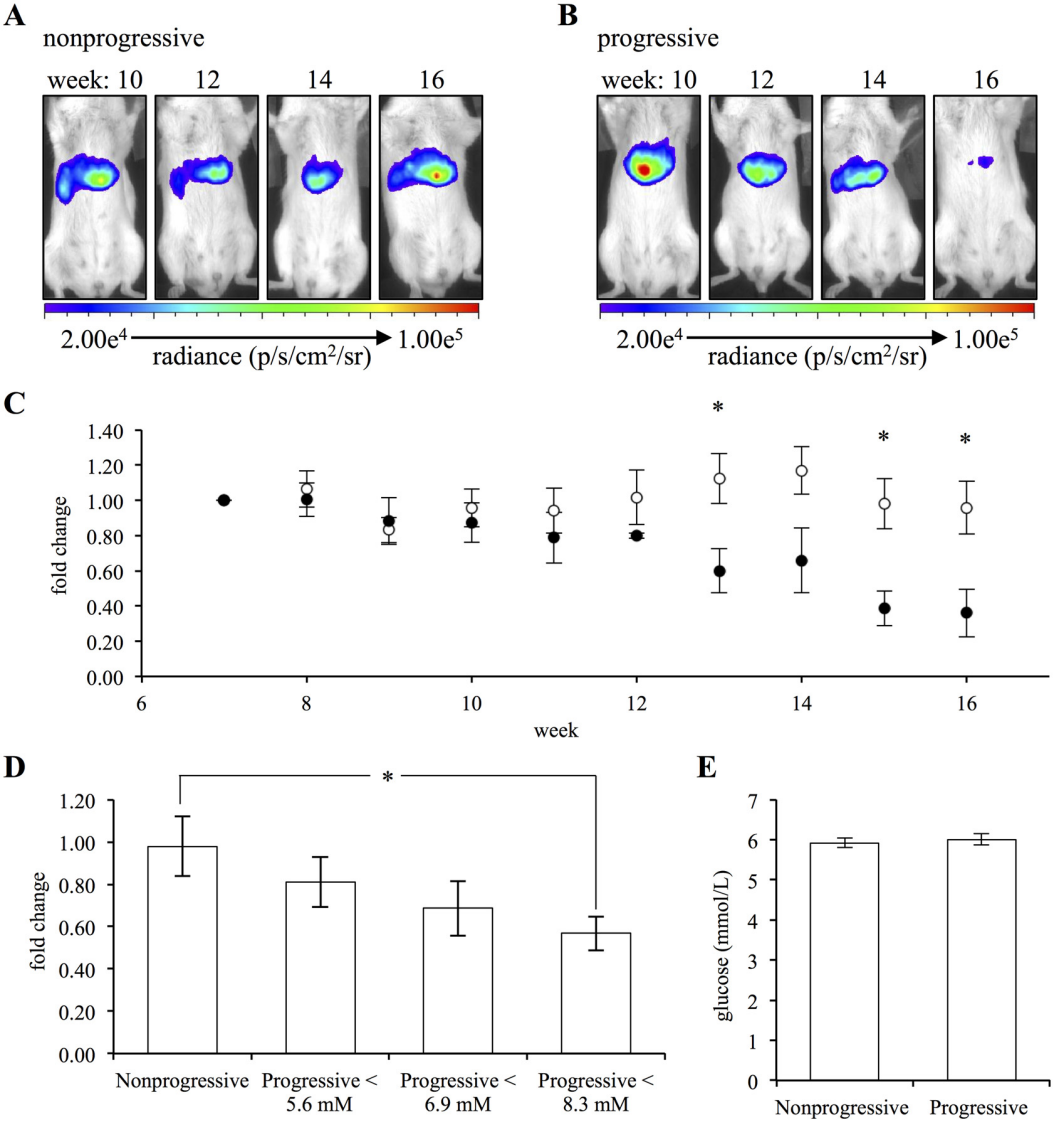
Superoxide anion concentration, as detected with coelenterazine, serves as an *in vivo* reporter of beta-cell mass. (A) Images of a representative nonprogressive mouse at 10, 12, 14 and 16 weeks of age. (B) Images of a representative progressive mouse at 10, 12, 14 and 16 weeks of age. The progressive mouse was normoglycemic at the time of the images. (C) The signal intensities for the two groups of mice, nonprogressive (white) (n = 8) and progressive (black) (n = 7), for a given age normalized to the first day of imaging for the corresponding mouse and expressed as the fold change in signal intensity. (D) The signal intensities for the nonprogressive mice during the last week of the study normalized to the first day of imaging for the corresponding mouse and expressed as the fold change in signal intensity. The signal intensities for progressive mice for the last day of the study during which their fasting blood glucose levels were not exceeding 5.6, 6.9 or 8.3 mmol/L normalized to the first day of imaging for the corresponding mouse and expressed as the fold change in signal intensity. (E) The mean of the fasting blood glucose levels that were less than 8.3 mmol/L for nonprogressive and progressive mice. (Error bars represent the mean ± SEM, and * denotes a significant difference between the groups of P < 0.039.)

For analysis, each NOD mouse was defined as either nonprogressive or progressive. A NOD mouse that did not develop hyperglycemia throughout the entire study period was defined as a nonprogressive. A progressive mouse developed hyperglycemia, defined as a blood glucose level of greater than 22 mmol/L, at some point during the length of the study. By completion of the study, 47% of the NOD mice had developed hyperglycemia; the age of disease development varied for each mouse. Over the course of monitoring, the nonprogressive mice did not demonstrate any change in beta-cell superoxide anion concentrations as detected with coelenterazine (Fig 7A). In contrast, the progressive mice showed a decrease in signal prior to the onset of hyperglycemia (Fig 7B). There was a significant difference in the signal intensities of progressive and non-progressive mice at 13, 15 and 16 weeks of age (Fig 7C). Additionally, there was a significant negative correlation between the reduction in coelenterazine chemiluminescent signal intensity and the age of the mouse for the progressive group (R^2^ = 0.9167); however, there was no such correlation in the nonprogressive group (R^2^ = 0.0609) (S9A and S9B Fig).

As type I diabetes disease progression is unique to each individual NOD mouse and not dependent upon mouse age, we aimed to define an additional stratification method for the progressive population [54]. When analyzed in regard to blood glucose levels, hyperglycemic mice had a significant 62 ± 6% decrease in coelenterazine signal intensity over the course of the study (P = 9.85x10^-11^); no such decrease was observed for normoglycemic mice (S10 Fig). Although informative, to better evaluate if superoxide anion was serving as a *predictive* biomarker for disease susceptibility, we compared the coelenterazine signal intensities from nonprogressive and normoglycemic-progressive mice. Progressive mice were further stratified based upon fasting blood glucose levels; groups were composed of the image last taken *before* the fasting blood glucose level exceeded 5.6, 6.9 or 8.3 mmol/L. Progressive mice had a clear reduction in signal intensity relative to nonprogressive mice for each subsequent higher fasting blood glucose level (Fig 7D). The fold change in signal intensity for the last image before the fasting blood glucose level exceeded 8.3 mmol/L was significantly lower for progressive mice compared to nonprogressive mice (P = 0.0390). More importantly, when all the fasting blood glucose of less than 8.3 mmol/L for the nonprogressive and progressive mice were averaged there was no significant difference between the two groups, indicating that coelenterazine chemiluminescence was an earlier indicator of disease progression than fasting blood glucose levels (Fig 7E). These results demonstrate that beta-cell superoxide anion, as detected with coelenterazine, serves as a measure of beta-cell mass and, as such, is an early predictive biomarker of diabetes mellitus.

## Discussion

The goal of this work was to evaluate the dynamic changes in superoxide anion concentration in cells and organisms in response to environmental and pathological stimuli. We approached this by validating coelenterazine as a high-throughput, dynamic reporter of superoxide anion concentrations and by applying this method both *in vitro* and *in vivo*. Our results argue that cells dynamically maintain superoxide anion homeostasis by regulating its production or removal depending upon the environment. Additionally, it suggests that *acute* physiological and even pathological hyperglycemia does not result in an overabundance of superoxide anion but, actually an *acute* decrease in concentrations. This decrease is followed by a rapid increase in the rate of superoxide anion accumulation in cells under hyperglycemic conditions. Finally, our findings suggest that chronic, pathological hyperglycemia can result in elevated concentrations of superoxide anion within two hours of continuous exposure. We also identify superoxide anion as a biomarker for beta-cell mass *in vivo* and demonstrate its ability to predict susceptibility to diabetes mellitus.

### Dynamic, longitudinal quantification of superoxide anion *in vitro*

Coelenterazine chemiluminescence was a useful reporter for cellular superoxide anion concentrations. However, it is important to note that coelenterazine, like all reactive oxygen species reporters, suffers from some limitations. Firstly, coelenterazine has a high auto-oxidation rate once in solution, and this rate is dependent upon the solution conditions [30]. As such, it is necessary to employ proper controls, stringent experimental guidelines and to limit data interpretation to only the data obtained within a given (plate) experiment. Additionally, coelenterazine is not specific for superoxide anion, but can also react with peroxynitrite [22,26]. Although peroxynitrite did not appear to be contributing significantly to the signal observed in our experiments, this is an important consideration when using coelenterazine as a reactive oxygen species reporter.

In our work coelenterazine proved to be detecting primarily superoxide anion concentrations in our cell assays, specifically that produced during oxidative phosphorylation when under static, basal conditions. These results are consistent with prior literature which has demonstrated that the majority of cellular superoxide anion originates from oxidative phosphorylation [31–33]. The use of coelenterazine as a superoxide anion reporter has several advantages over currently used methods. Fluorescent reporters have been primarily optimized for microscopy, which limit the study of superoxides to *in vitro* or *ex vivo* analyses with small fields of view, and, since the fluorescent reporters are accumulation probes and are unable to detect reductions in superoxide anion concentrations, studies requiring dynamic quantifications with fine-temporal resolution are not feasible. This is particularly true when decreases in superoxide anion concentrations are to be studied [16–18]. Chemiluminescent reporters, such as coelenterazizne enable dynamic, high-throughput monitoring of superoxide anion concentrations. Of fundamental importance is that chemiluminescent reporters are not accumulation probes like common superoxide anion reporters. A photon is released for detection upon reaction and, once that photon is released, no further signal can be achieved from that specific pair of molecules. As such, the signal detected is for the quantity of superoxide anion present within the exposure time that a given image is taken. This enables the visualization of small changes, including increases and decreases, in superoxide concentrations with fine temporal resolution of entire populations of cells. With the exception of coelenterazine, all other chemiluminescent reporters of reactive oxygen specis are limited to detecting extracellular superoxide anion concentrations [23,24,27]. As the pathological consequences of dysregulation in reactive oxygen species are often intracellular, the most relevant fluctuations in superoxide anion concentrations are those that occur within the cell. Collectively, the unique features of coelenterazine make it a valuable tool for the quantification of the dynamic response of intracellular superoxide anion concentrations to physiological and pathological cues.

### Glucose-stimulated reduction in superoxide anion concentrations maintains homeostasis

The use of coelenterazine for quantification of superoxide anion concentration enabled us to observe the acute response of cells to a range of normo- and hyperglycemic conditions. Previous research has primarily focused on the affects of chronic, supra-pathological glucose exposures; typical exposures include concentrations in excess of 30 mmol/L glucose for several consecutive days prior to the detection of superoxide anion [34,37–39]. Our aim was to examine the effects of transient post-prandial hyperglycemia, which occurs in both healthy individuals and, more severely, in the insulin resistant. Our results argue that there is an acute and transient decrease in superoxide anion concentrations in response to hyperglycemia.

The acute decrease in response to hyperglycemia was followed by a rapid increase in the rate of superoxide accumulation by cells in hyperglycemic versus normoglycemic conditions. This increased rate of accumulation is consistent with prior works and may provide a clue as to the cause for the discrepancy between our work and others in regards to the earlier acute response phase. More common superoxide anion reporters are characterized by an accumulation of signal intensity over time. As such, these reporters cannot quantify reductions in superoxide anion concentration and suffer from high background due to the accumulation of signal pre-intervention, reducing their dynamic range. This also limits their usefulness for detecting finite changes with high temporal resolution. As such, the decrease in coelenterazine signal intensity that we observed within the first 15 minutes would appear as a slight reduction in the slope of probe accumulation over the same time span. Given the accumulated background signal, such an effect would be easily masked and harder to quantify. Our ability to detect an early, rapid acute decrease in superoxide concentrations is likely due to two main factors: the highly-dynamic, chemiluminescent nature of coelenterazine and our choice to longitudinal and continuously monitor cells immediately post-glucose addition.

The early, rapid decrease in superoxide anion concentrations in response to hyperglycemia does not preclude the notion that chronic hyperglycemia results in oxidative damage to tissues. Our results support that during chronic (≥ 2 hours), pathological hyperglycemia, the rate of superoxide anion accumulation begins to exceed its removal, resulting in higher concentrations of superoxide anion in some cell lines. We postulate that disruption in the early kinetics of the glucose response can promote greater concentrations of superoxide anion with reduced hyperglycemia exposure times; that a failure in the degree or speed of reduction in superoxide anion concentrations in the acute response increases the likelihood that the following increased rate of superoxide anion accumulation will exceed the newly achieved tolerance capacity of the cell. Indeed, one can ask if chronic hyperglycemia itself results in dysfunction of this homeostatic mechanism, and, if so, how might this relate to the oxidative damage observed in patients suffering from chronic hyperglycemia.

### Superoxide anion as a biomarker for beta-cell mass

Our *in vivo* studies revealed that the insulin-secreting cells, the beta cells, of the pancreas have significantly higher superoxide anion concentrations than other body tissues and that the concentration fluctuates in response to physiological cues. This result is supported by the knowledge that beta cells have low concentrations of scavenger enzymes and utilize reactive oxygen species as signaling molecules during the process of insulin secretion [45–49]. Since a reduction in beta-cell mass is a prerequisite to the development of diabetes mellitus, we tested if superoxide anion could serve as a biomarker for beta-cell mass and, thereby, an indicator of disease susceptibility [52]. Molecular imaging targets specific to the beta-cell are actively being sought as a diagnostic aid. Our results indicate that detection of superoxide anion may provide such a target; however, significant advances in imaging techniques would be required before it can be determined if these findings apply to human beta cells as well.

Reactive oxygen species are thought to play a role in beta cell apoptosis during type I diabetes [57]; however, we did not observe an increase in chemiluminescence in NOD mice prior to the onset of clinical disease. There are several possible reasons for this: each NOD mouse develops disease at a unique time, we imaged once weekly, and we averaged across the population. As such, any increase in signal intensity may not have been uniform and/or significant enough for detection. Additionally, the coelenterazine chemiluminescence observed *in vivo* represents the average superoxide anion concentration among all beta cells; however, individual islets of Langerhans may be undergoing different stages of disease and an absence of an increase in macroscopic coelenterazine chemiluminescence cannot be used as an indicator of the microscopic cellular responses of different cell population subsets.

Collectively, our research demonstrated that coelenterazine imaging is a high-throughput method for quantifying the dynamics of superoxide anion concentration, emphasizing the role of superoxide anion homeostasis in the cellular response to glucose and providing new fundamental insights into possible mechanisms for dysregulation–as pathologies may be associated with a failure in the dynamic regulation of superoxide anion. It also demonstrated, *in vivo*, the high superoxide concentration of beta cells and its potential as a biomarker for beta-cell mass. Collectively, superoxide anion was revealed to have a dynamic and important place in cellular and organismal glucose metabolism.

## Materials and Methods

### Materials

Coelenterazine was purchased from Nanolight (#303). PEG-SOD (pegylated superoxide dismutase #S9549), PEG-CAT (pegylated catalase #C4963), uric acid (#U2625), d-glucose (#G8270), 4-OHCA (alpha-cyano-4-hydroxycinnamic acid #M7033), AOAC (aminooxyacetic acid #C13408), GKA50 (#L2630), Ranolazine (#R6152) and streptozotocin (#S0130) were all purchased from Sigma Aldrich.

### Cell Culture

Cell lines were cultured in RPMI-1640 with 10% fetal bovine serum (Invitrogen), 2 mmol/L glutamine, 100 U/mL penicillin and 100 mg/mL streptomycin. INS-1 cell media was additionally supplemented with 50 umol/L 2-mercaptoethanol. HeLa and RIN-5F cells were obtained from ATCC. INS-1 cells were obtained from AddexBio. The firefly luciferase-expressing HeLa cell line were derived from a stable line used previously in our lab [58].

### Imaging Cells in Culture

Coelenterazine: Cell lines were split 48-hours prior to the experiment to achieve a final confluency of approximately 60% the day of the experiment. The day of the experiment, cells were collected by trypsinization. Following collection, cells were washed twice in PBS and resuspended in Krebs buffer (140 mmol/L NaCl, 30 mmol/L Hepes, 4.6 mmol KCl, 1 mmol/L MgSO_4_, 0.15 mmol/L Na_2_HPO_4_, 5 mmol/L NaHCO_3_ and 2 mmol/L CaCl_2_) supplemented with 4 mmol/L D-glucose. Unless stated otherwise, native coelenterazine at a final concentration of 10 μmmol/L was used for all imaging. When cells were both imaged and treated, cells were incubated in coelenterazine for 15 minutes prior to treatment. HeLa cells were treated with PEG-SOD, PEG-CAT and uric acid [59–61]. Additionally, HeLa cells were treated with metabolic pharmaceuticals as previously described [34,35,62]. All samples were completed in triplicate. All the samples shown in an individual figure category were completed within the same well plate during the same imaging session to exclude inter-experimental variability during data interpretation. The total flux (p/s) was quantified for each well using the Living Image software and expressed as the total flux (p/s) or the fold change relative to the control.

Dihydroethidium: HeLa cells were split 48-hours prior to the experiment to achieve a final confluency of approximately 60% the day of the experiment. The day of the experiment, cells were collected by trypsinization. Following collection, cells were washed twice in PBS and resuspended at a concentration of 5x10^6^ cells/mL in Krebs buffer (140 mmol/L NaCl, 30 mmol/L Hepes, 4.6 mmol KCl, 1 mmol/L MgSO_4_, 0.15 mmol/L Na_2_HPO_4_, 5 mmol/L NaHCO_3_ and 2 mmol/L CaCl_2_) supplemented with 4 mmol/L D-glucose. 5x10^5^ HeLa cells were added to each well of a 96-well black plate. Dihdyroethidium staining was completed as previously described [63]. Briefly, dihydroethidium was suspended in dimethyl sulfoxide (DMSO) to a concentration of 10 mM. Dihydroethidium or equal volume of vehicle control was added to cell suspensions to a final concentration of 5 uM and incubated for thirty minutes protected from light. Baseline (-5 minute) fluorescence was detected using the Biotek^®^ Synergy 4 plate reader using an excitation of 490 nm and an emission of 590 nm. Cell suspension glucose concentrations were adjusted to a final concentration of 4 mmol/L or 20 mmol/L and fluorescence was sequentially detected at 5-minute intervals.

### Fluorescence Microscopy

Whole, excised pancreas was immediately incubated in PBS with 1% FBS and 20 μmmol/L coelenterazine for one hour at 37°C protected from light. The tissue was washed twice for 20 minutes in PBS and then fixed with 10% formalin for 20 minutes. Following fixation, the pancreas was washed twice with PBS with 1% FBS. The pancreas was then incubated with anti-insulin antibody (Abcam^®^ ab6995) at a 1/1000 dilution for one hour, washed twice washed twice with PBS with 1% FBS, and incubated with goat anti-mouse IgG secondary Alexa Fluor^®^ 750 conjugate (Thermo Fisher Scientific #A21037) for an additional hour. The tissue was washed three times with PBS, 1% FBS for 20-minutes each. The whole pancreas was imaged using the using the EVOS^®^ Cell Imaging System. Coelenterazine fluorescence was detected using the green-fluorescent (GFP) light cube (excitation: 470/22 nm and emission: 510/42 nm) and the Alexa Fuor^®^ 750 conjugate detected using the Cy7 light cube (excitation: 710/40 nm and emission: 775/45 nm).

### Ethics Statement

All animal studies were completed in accordance with the Guide for the Care and Use of Laboratory Animals of the National Institutes of Health and approved by the Institutional Animal Care and Use Committee of Stanford University.

### *In Vivo* Imaging

To minimize coelenterazine auto-oxidation, coelenterazine was resuspended in 100% ethanol to a concentration of 5mg/mL [30]. Individual aliquots of coelenterazine were stored on dry ice protected from light until immediately prior to imaging. At the time of imaging, an aliquot was diluted to a final concentration of 1mg/mL in 5% glucose solution. Mice were imaged individually and anesthetized using isoflurane inhalant anesthetic. Unless stated otherwise, mice were administered 5mg/kg native coelenterazine intravenously. Mice were immediately imaged using the IVIS Spectrum optical imaging platform in the Stanford University Small Animal Imaging Facility. The data was quantified using the Living Image software. Unless stated otherwise, a region of interest (ROI) was selected using the software’s auto-draw function with a threshold of 50%. Data was presented as the average radiance (p/s/cm^2^/sr) or as the fold change relative to the corresponding baseline image.

#### Streptozotocin mouse model

Eight, male, eight-week-old MIP-Luc-VU mice were administered 200 mg/kg streptozotocin or vehicle control as previously described [64]. Blood glucose levels were monitored using the One Touch Ultra glucose meter.

#### NOD mouse model

Fifteen, female, NOD mice were purchased from Charles River at five-weeks-of-age and imaged weekly.

### Statistical Analysis

Data was presented as the mean ± SEM. Significance was determined by the Student’s 2-tailed T test and defined as P ≤ 0.05.

## Supporting Information

S1 Fig Coelenterazine chemiluminescence and 2-hydroxyethidium fluorescence positively correlate. Correlation graph of coelenterazine chemilumnesecence computed as the cumulative area under the curve versus 2-hydroxyethidium fluorescence as fluorescence intensity. There was a significant (P < 0.0001) positive correlation (R^2^ = 0.6753) between the two reporters (n = 3).
(TIFF)

S2 Fig Coelenterazine administration in vivo results in an abdominal and thoracic signal with varying imaging kinetics. (A) A mouse was administered 10 mg/kg native coelenterazine and imaged sequentially in 1-minute intervals for 20 minutes. (B) Quantification of the chemiluminescent signal from an ROI selected over the thoracic region or abdominal region (n = 1).
(TIFF)

S3 Fig Administration of several coelenterazine analogs results in a similar chemiluminescent abdominal signal. (A) Mice were intravenously administered 25 ug of a coelenterazine analog and imaged immediately for 1, 5-minute exposure. (B) Quantification of the chemiluminescent signal from an ROI auto-selected for the area of greatest signal intensity (n = 3). (Error bars represent the mean ± SEM.)
(TIFF)

S4 Fig Imaging with coelenterazine at various time points throughout the day results in variations in chemiluminescent signal intensity. (A) Images of a representative animal from each time point administered 5 mg/kg of native coelenterazine. (B) Quantification of the chemiluminescent signal from an ROI auto-selected for the area of greatest signal intensity for each time point of imaging (n = 3). (Error bars represent the mean ± SEM.)
(TIFF)

S5 Fig Fasted mice have a reduced chemiluminescent signal compared to control mice. (A) Mice were fasted for 6 hours or not (control) and imaged with 5 mg/kg of native coelenterazine. (B) Quantification of the chemiluminescent signal from an ROI auto-selected for the area of greatest signal intensity (n = 5). (Error bars represent the mean ± SEM.)
(TIFF)

S6 Fig The absorption and emission spectra of coelenteramide. (A) Absorption spectrum of coelenteramide. (B) Emission spectrum of coelenteramide upon excitation at 420 nm.
(TIFF)

S7 Fig Ex vivo coelenteramide-mediated fluorescence is not chemiluminescence or autofluorescence. (A) Detection of chemiluminescence from the tissues in Fig 5*E* with restriction of the emission as in Fig 5*E*. (B) Fluorescent imaging of the pancreata and spleens from mice administered 5 mg/kg coelenterazine or vehicle control. (C) Quantification of the fluorescent signal from ROIs selected for the pancreata and spleens (n = 4). (Error bars represent the mean ± SEM.)
(TIFF)

S8 Fig The reduction in coelenterazine signal intensity in streptozotocin-treated mice is not a consequence of hyperglycemia. (A) The coelenterazine chemiluminescent signal produced from streptozotocin-treated (STZ) mice during hyperglycemia (white) and following insulin therapy (grey). Control (Control) mice imaged on the corresponding days but not administered insulin (n = 4). (B) Blood glucose levels of streptozotocin mice without insulin therapy (Untreated) and following insulin therapy immediately prior to imaging (Treated). (Error bars represent the mean ± SEM, and * denotes a significant difference between the groups of P = 0.0002.)
(TIFF)

S9 Fig Superoxide anion concentration, as detected with coelenterazine, negatively correlates with the age of progressive mice. (A) The coelenterazine chemiluminescent signal intensities for the nonprogressive mice of a given age normalized to the first day of imaging for the corresponding mouse and expressed as the fold change in signal intensity. (B) The coelenterazine chemiluminescent signal intensities for the progressive mice of a given age normalized to the first day of imaging for the corresponding mouse and expressed as the fold change in signal intensity. (Error bars represent the mean ± SEM.)
(TIFF)

S10 Fig Hyperglycemic NOD mice have a reduced chemiluminescent signal compared to normoglycemic NOD mice. The fold change in signal intensity of each image taken during normoglycemia (n = 199) or hyperglycemia (n = 23). Each image was normalized to the first day of imaging for the corresponding mouse and expressed as the fold change in signal intensity. (Error bars represent the mean ± SEM, and * denotes a significant difference between the groups of P < 0.035.)
(TIFF)
