# Supplementary figures and images for: Chemiluminescence Imaging of Superoxide Anion Detects Beta-Cell Function and Mass
Source: PLoS One. 2016 Jan 11;11(1):e0146601. doi: 10.1371/journal.pone.0146601 (PMC4709142; doi:10.1371/journal.pone.0146601)

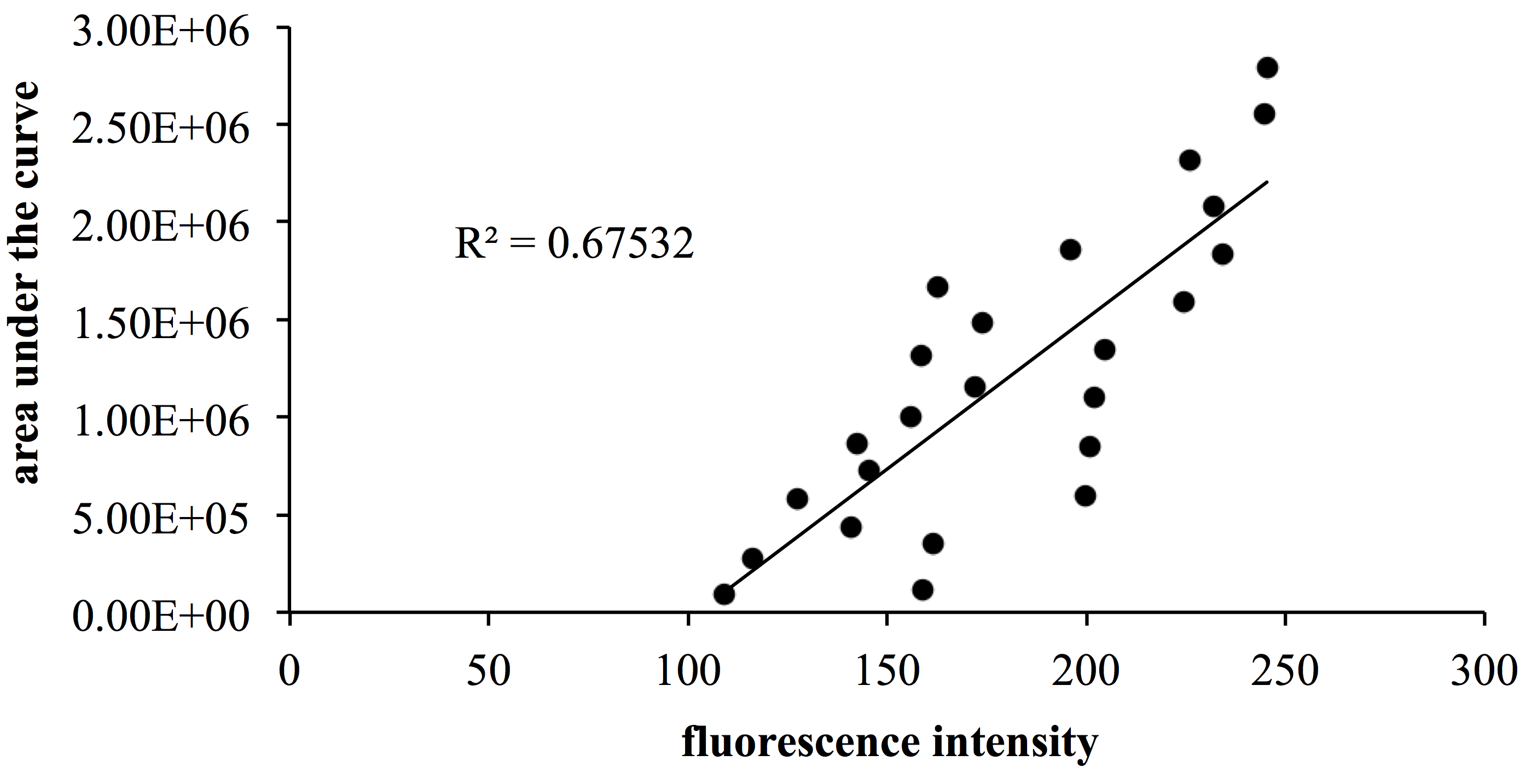

Supplement: S1 Fig — Correlation graph of coelenterazine chemilumnesecence computed as the cumulative area under the curve versus 2-hydroxyethidium fluorescence as fluorescence intensity. There was a significant (P < 0.0001) positive correlation (R2 = 0.6753) between the two reporters (n = 3). (TIFF) [file pone.0146601.s001.tiff]

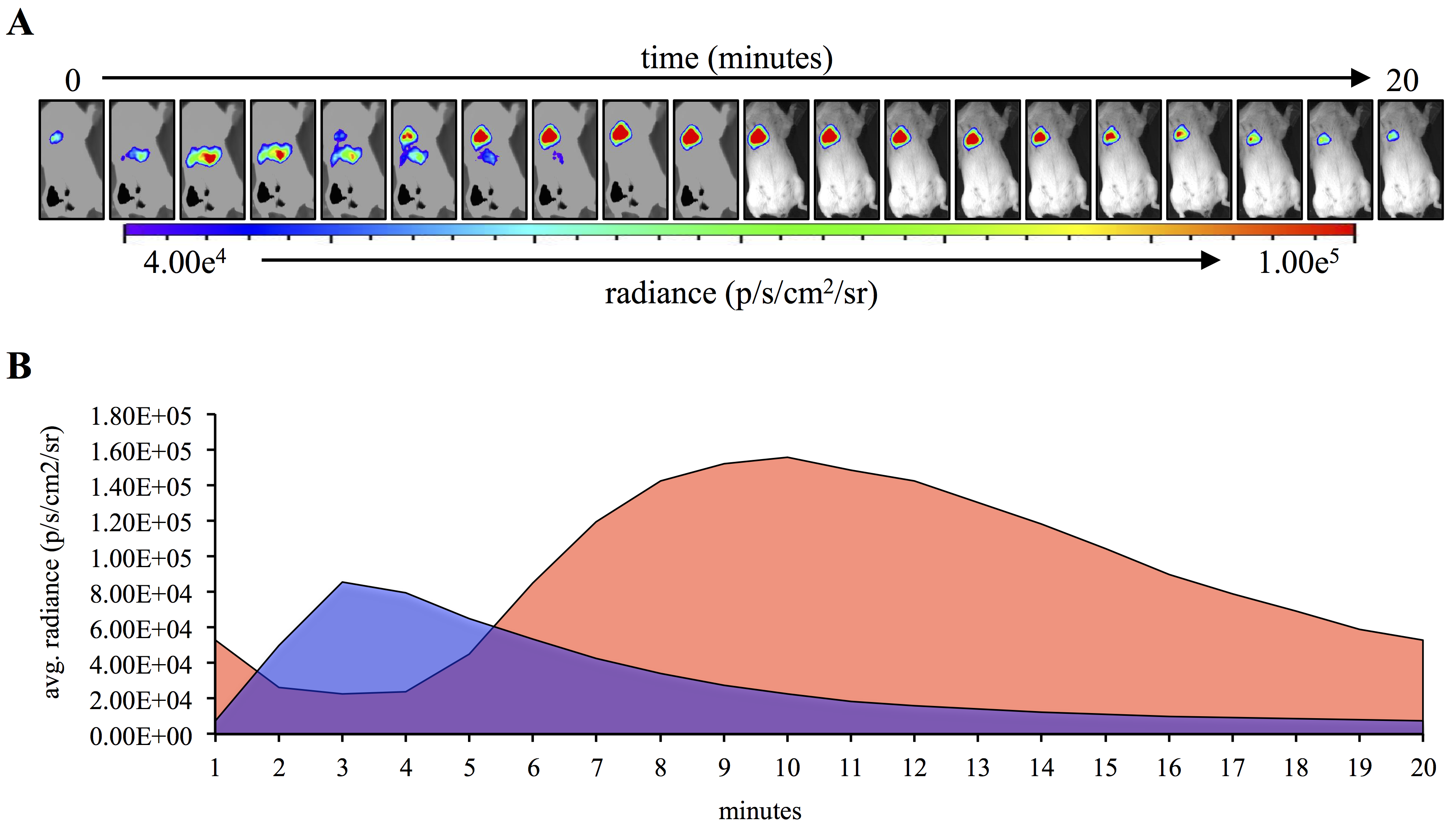

Supplement: S2 Fig — (A) A mouse was administered 10 mg/kg native coelenterazine and imaged sequentially in 1-minute intervals for 20 minutes. (B) Quantification of the chemiluminescent signal from an ROI selected over the thoracic region or abdominal region (n = 1). (TIFF) [file pone.0146601.s002.tiff]

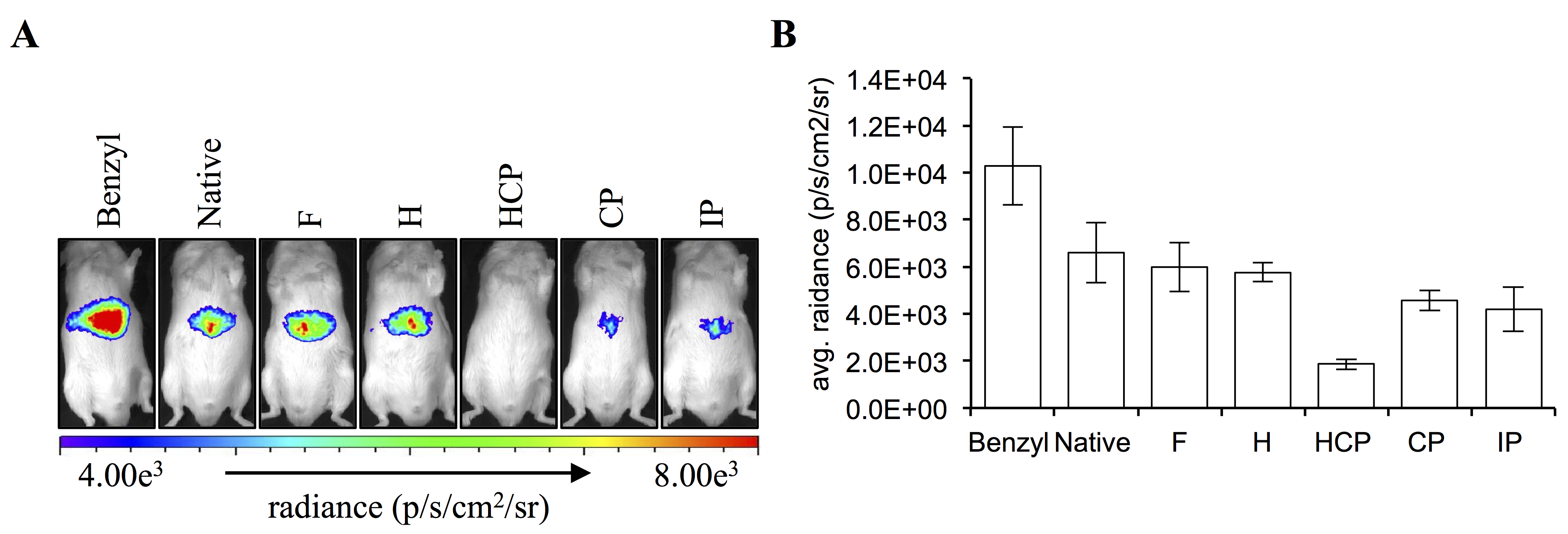

Supplement: S3 Fig — (A) Mice were intravenously administered 25 ug of a coelenterazine analog and imaged immediately for 1, 5-minute exposure. (B) Quantification of the chemiluminescent signal from an ROI auto-selected for the area of greatest signal intensity (n = 3). (Error bars represent the mean ± SEM.) (TIFF) [file pone.0146601.s003.tiff]

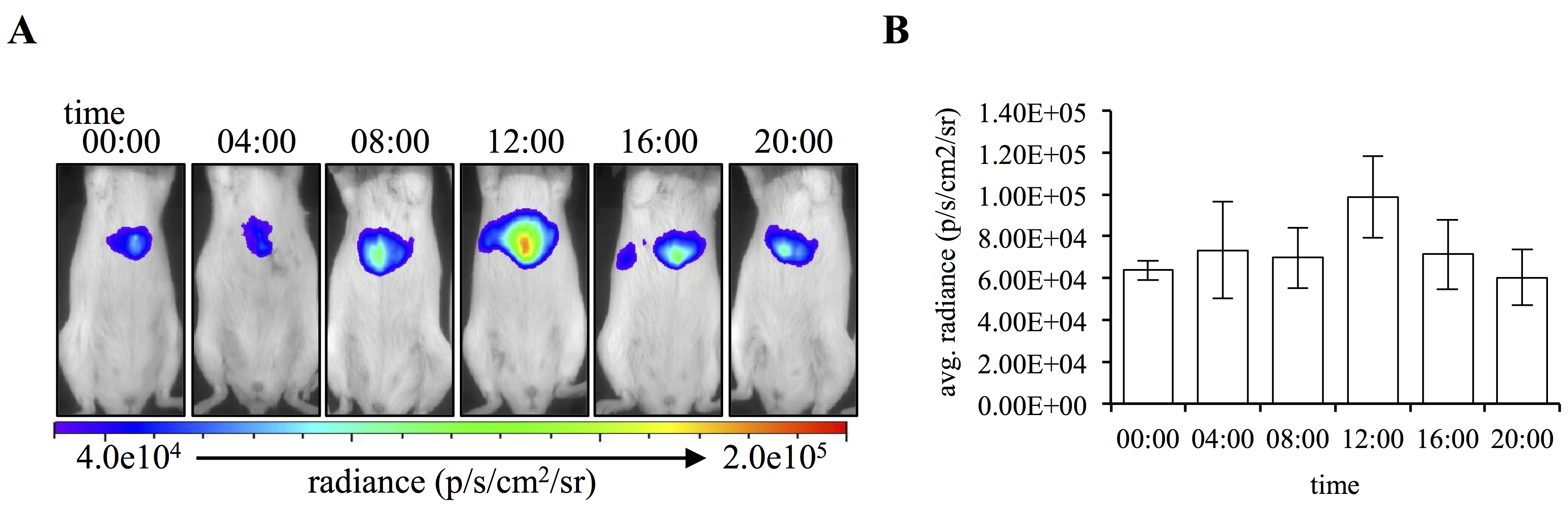

Supplement: S4 Fig — (A) Images of a representative animal from each time point administered 5 mg/kg of native coelenterazine. (B) Quantification of the chemiluminescent signal from an ROI auto-selected for the area of greatest signal intensity for each time point of imaging (n = 3). (Error bars represent the mean ± SEM.) (TIFF) [file pone.0146601.s004.tiff]

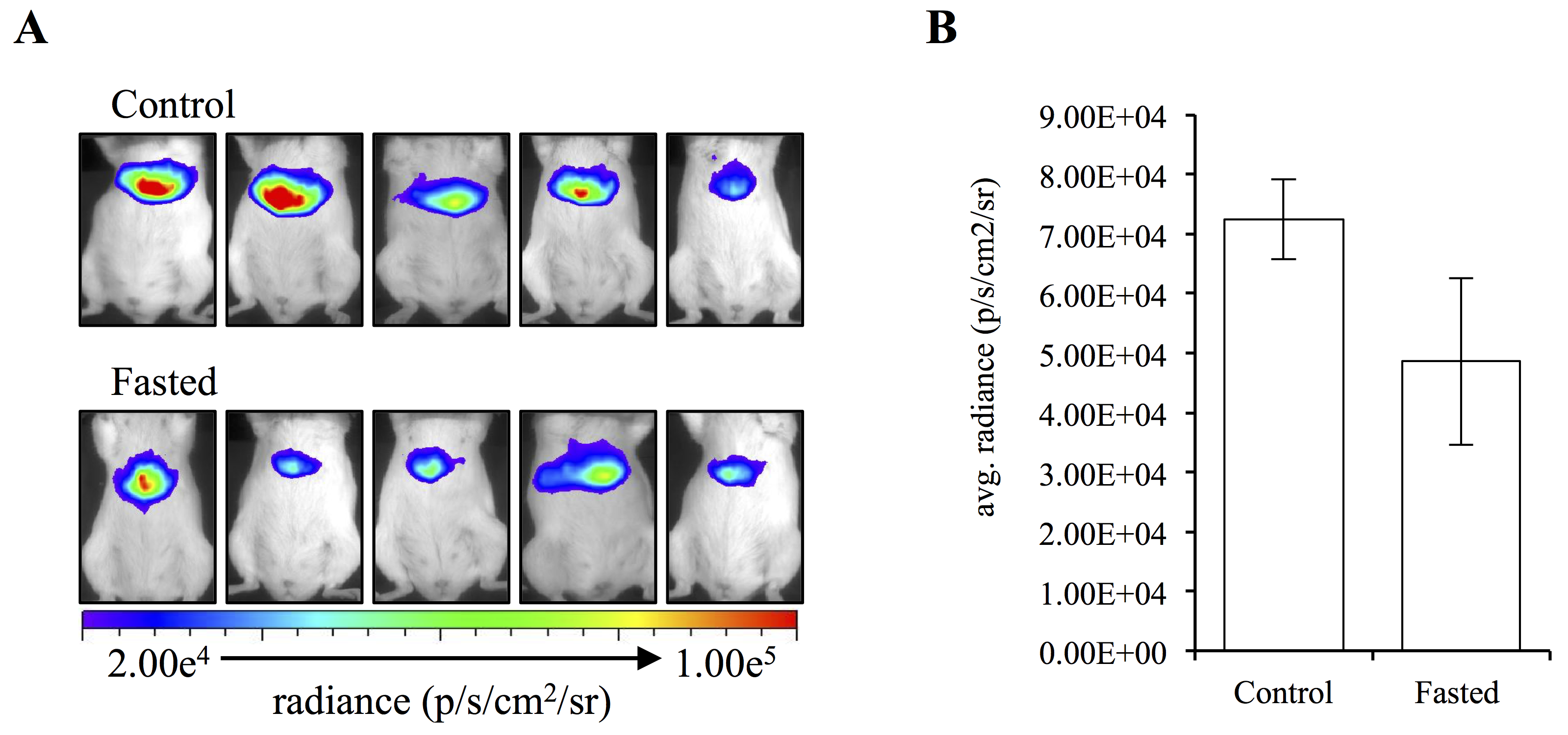

Supplement: S5 Fig — (A) Mice were fasted for 6 hours or not (control) and imaged with 5 mg/kg of native coelenterazine. (B) Quantification of the chemiluminescent signal from an ROI auto-selected for the area of greatest signal intensity (n = 5). (Error bars represent the mean ± SEM.) (TIFF) [file pone.0146601.s005.tiff]

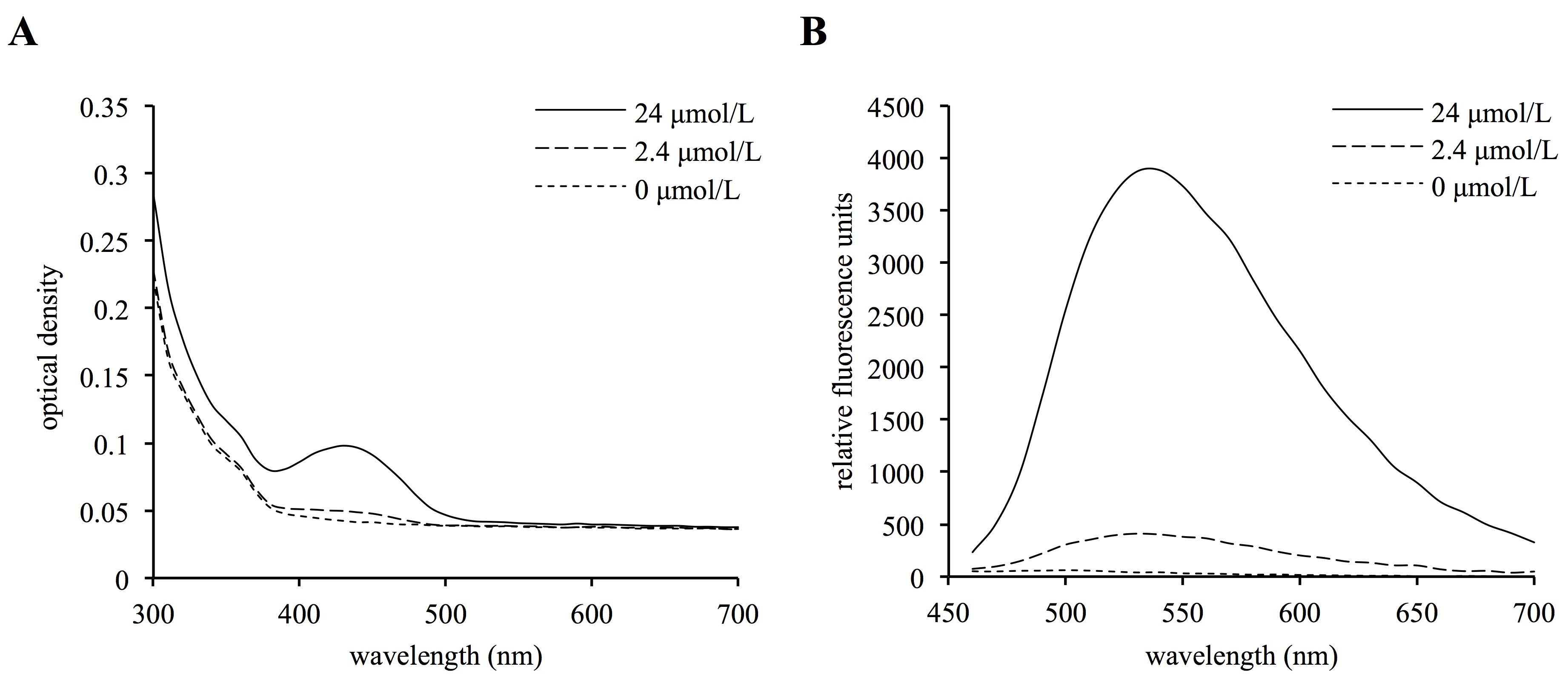

Supplement: S6 Fig — (A) Absorption spectrum of coelenteramide. (B) Emission spectrum of coelenteramide upon excitation at 420 nm. (TIFF) [file pone.0146601.s006.tiff]

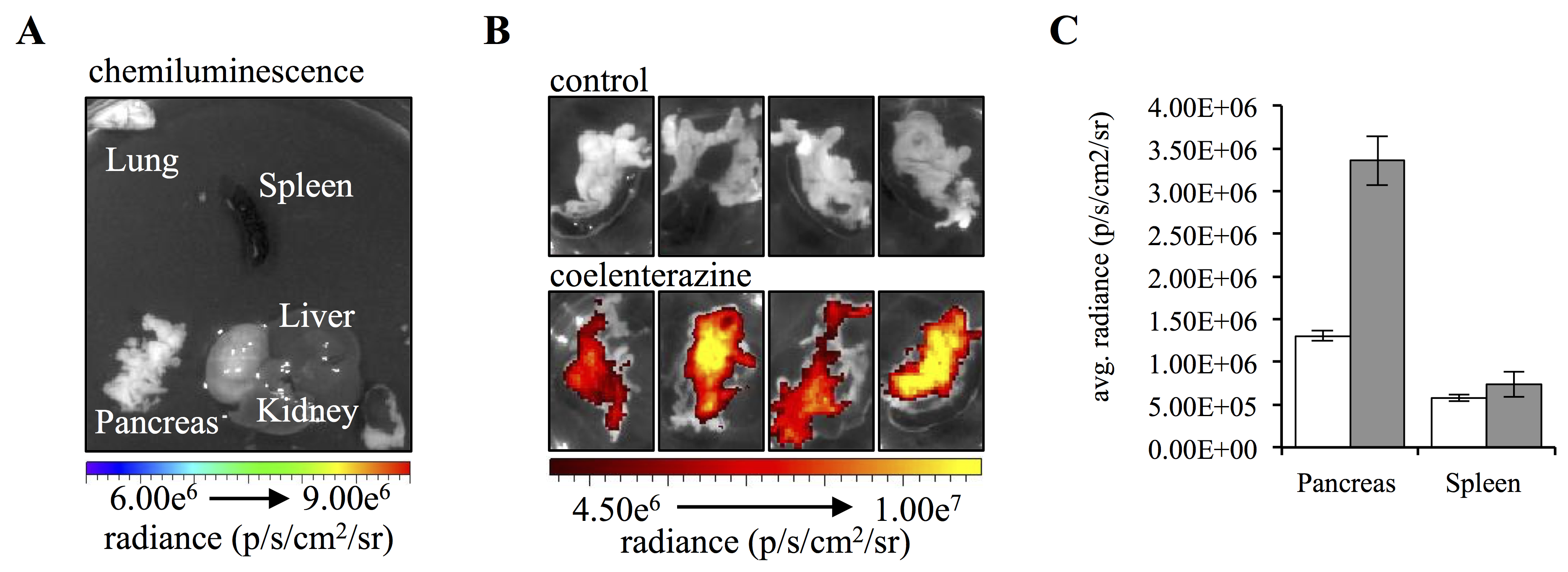

Supplement: S7 Fig — (A) Detection of chemiluminescence from the tissues in Fig 5E with restriction of the emission as in Fig 5E. (B) Fluorescent imaging of the pancreata and spleens from mice administered 5 mg/kg coelenterazine or vehicle control. (C) Quantification of the fluorescent signal from ROIs selected for the pancreata and spleens (n = 4). (Error bars represent the mean ± SEM.) (TIFF) [file pone.0146601.s007.tiff]

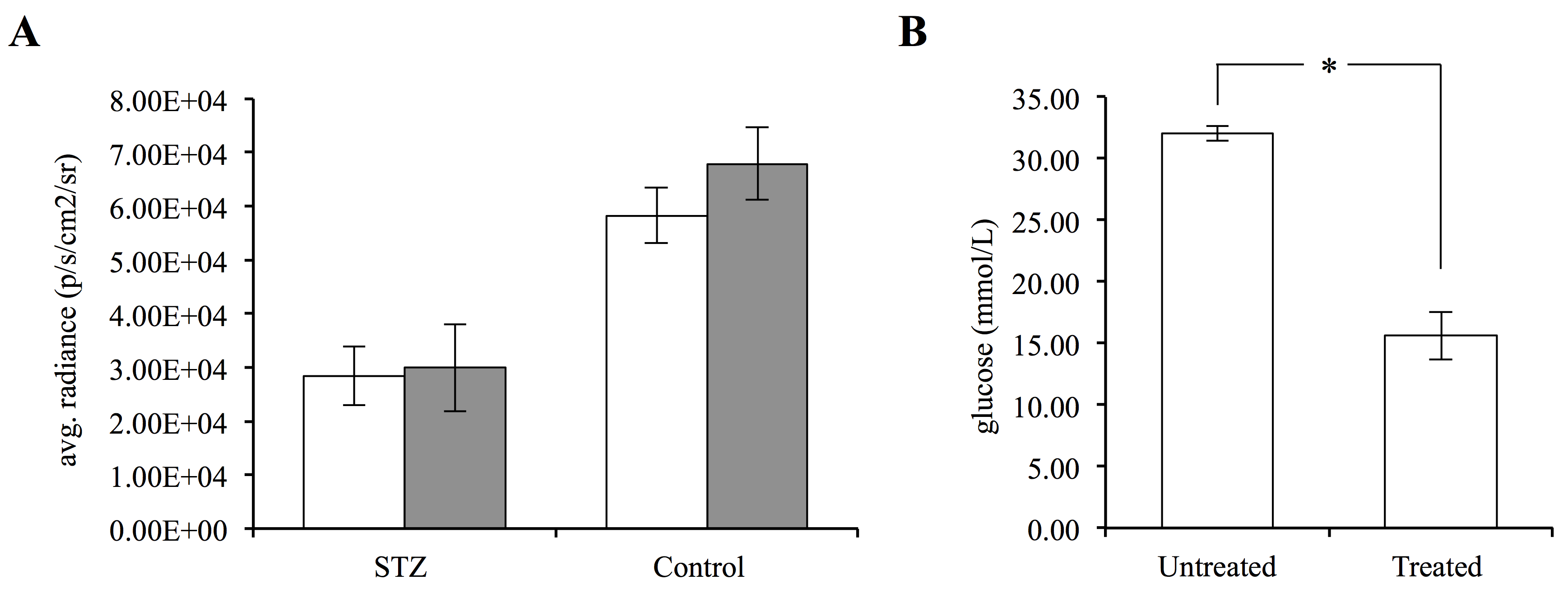

Supplement: S8 Fig — (A) The coelenterazine chemiluminescent signal produced from streptozotocin-treated (STZ) mice during hyperglycemia (white) and following insulin therapy (grey). Control (Control) mice imaged on the corresponding days but not administered insulin (n = 4). (B) Blood glucose levels of streptozotocin mice without insulin therapy (Untreated) and following insulin therapy immediately prior to imaging (Treated). (Error bars represent the mean ± SEM, and * denotes a significant difference between the groups of P = 0.0002.) (TIFF) [file pone.0146601.s008.tiff]

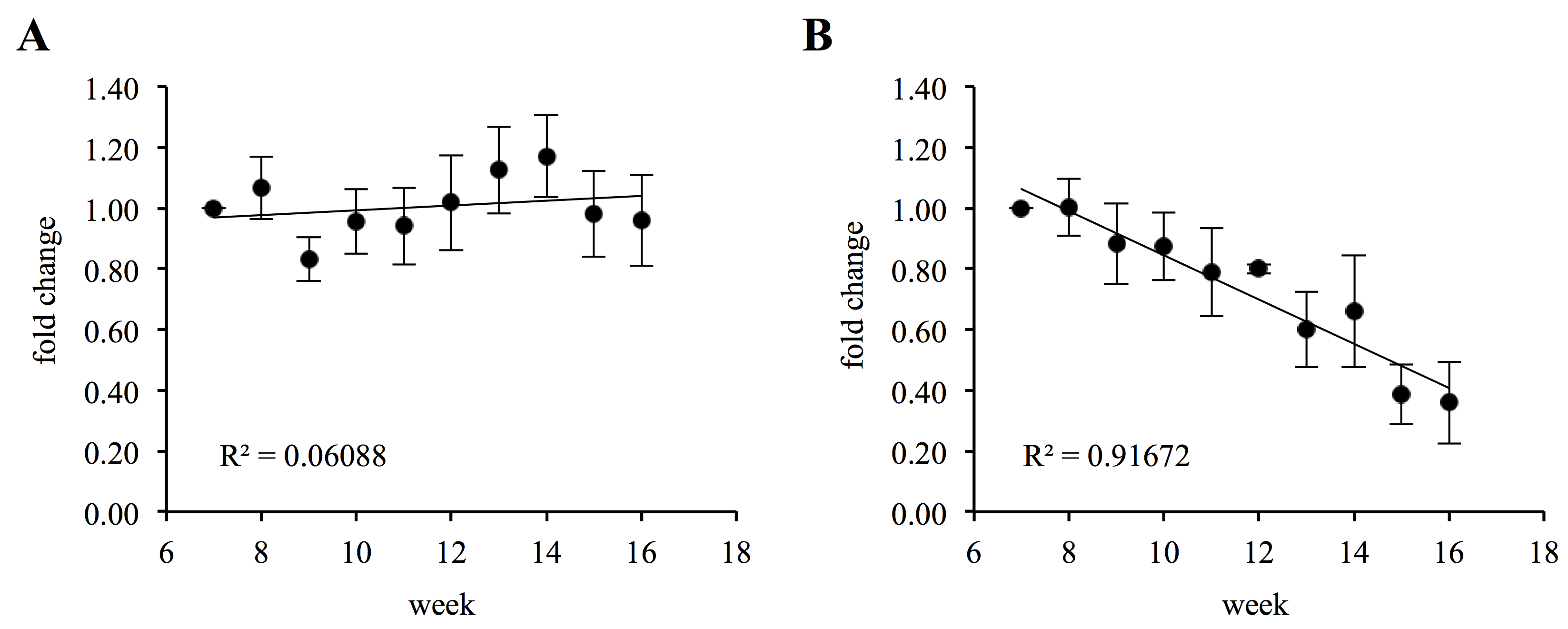

Supplement: S9 Fig — (A) The coelenterazine chemiluminescent signal intensities for the nonprogressive mice of a given age normalized to the first day of imaging for the corresponding mouse and expressed as the fold change in signal intensity. (B) The coelenterazine chemiluminescent signal intensities for the progressive mice of a given age normalized to the first day of imaging for the corresponding mouse and expressed as the fold change in signal intensity. (Error bars represent the mean ± SEM.) (TIFF) [file pone.0146601.s009.tiff]

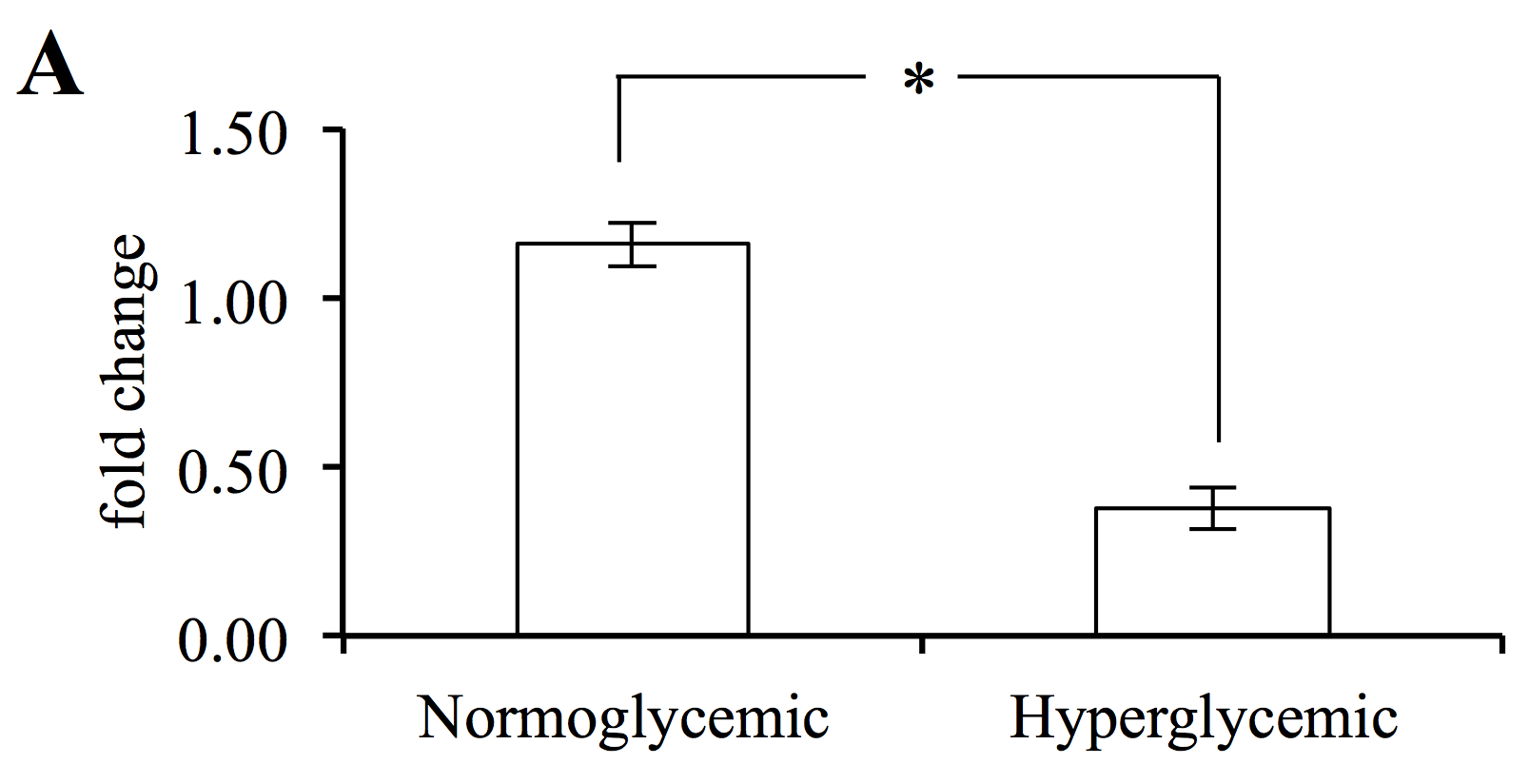

Supplement: S10 Fig — The fold change in signal intensity of each image taken during normoglycemia (n = 199) or hyperglycemia (n = 23). Each image was normalized to the first day of imaging for the corresponding mouse and expressed as the fold change in signal intensity. (Error bars represent the mean ± SEM, and * denotes a significant difference between the groups of P < 0.035.) (TIFF) [file pone.0146601.s010.tiff]
